# Supplementary material for: Effect of SARS-CoV-2 prior infection and mRNA vaccination on contagiousness and susceptibility to infection
Source: Nat Commun. 2023 Sep 6;14:5452. doi: 10.1038/s41467-023-41109-9 (PMC10482859; doi:10.1038/s41467-023-41109-9)
Supplement: Supplementary file 1 — Supplementary Information [file 41467_2023_41109_MOESM1_ESM.pdf]

Effect of SARS-CoV-2 prior infection and mRNA vaccination on contagiousness and susceptibility to infection

Supplementary material

Results 2
Testing of contacts 2
SAR as a function of the delay 3
Adjusted analysis with all immune category for index cases 4
Adjusted analysis with interaction term between contact immunity and propensity of contact to perform tests 6
Univariable and multivariable analysis 8
Adjusted analysis stratified per gender 10
Sensitivity analysis 12
Adjusted analysis using complete case analysis 12
Multivariable analysis 12
Multivariable analysis with interaction term 14
Adjusted analysis using the number of tests performed in the 180 days before the index-contact interaction as a proxy for testing 16
Adjusted analysis using periods with variant dominance of more than 90% 20
Misclassification of community cases 23
Misclassification of primary case 25
Misclassification of tertiary case 27
Methods 29
Background information 29
Evolution in Time of cases, tests and COVID19 deaths 29
Non-pharmaceutical interventions 30
Vaccination 31
Link between the index case and its contact 31
Symptoms 32
Personal vulnerability 32
Missing data 32
Population of Geneva 33
SARS-CoV-2 variants 33
Statistical analysis 34

Results

Testing of contacts

In this subsection we provide additional information concerning the testing of the contacts. Table S1 provide the age category of the contact and of the index for the contact who tested or not during the 10 days following their last encounter with the index case. Figure S1 provides the total number of tests performed by the contacts as a function of the delay in days since their last encounter with their index case.

	Did not test during the 10 days following encounter with index	Tested during the 10 days following encounter with index
N	65257	46417
Contact age (%)		
18-64	32131 (58.7)	30978 (66.8)
0-17	19675 (35.9)	13305 (28.7)
65+	2943 (5.4)	2110 (4.5)
Index age (%)		
18-64	48567 (74.4)	32777 (70.6)
0-17	13712 (21.0)	11627 (25.1)
65+	2975 (4.6)	2011 (4.3)

Table S1: Contact tested or not during the 10 days following their last encounter with the index case, by category of contact and index age

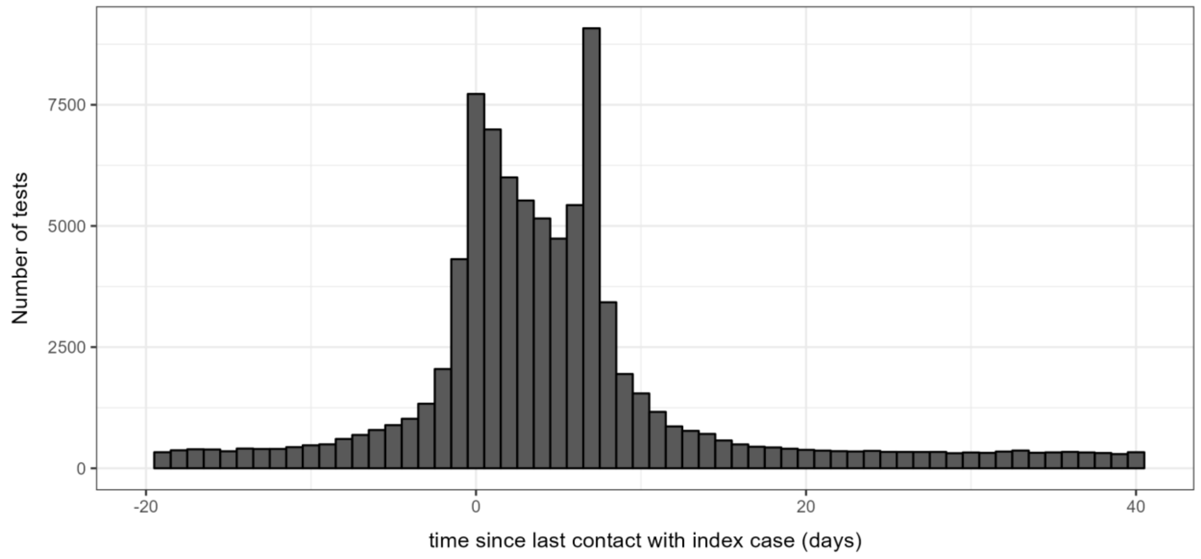

Figure S1: Total number of tests performed by the contacts as a function of the delay in days since their last encounter with their index case

The important peak in number of tests observed in Figure S1 at day 7 stems from the fact that from 8th February, 2021 to 31st of December 2021, the quarantine could be shortened with a negative test a day 7 (see methods in the main article).

SAR as a function of the delay

We present here the raw non stratified SAR as a function of the delay between the last encounter between the index and the contact and the positive test of the contact (figure S2)

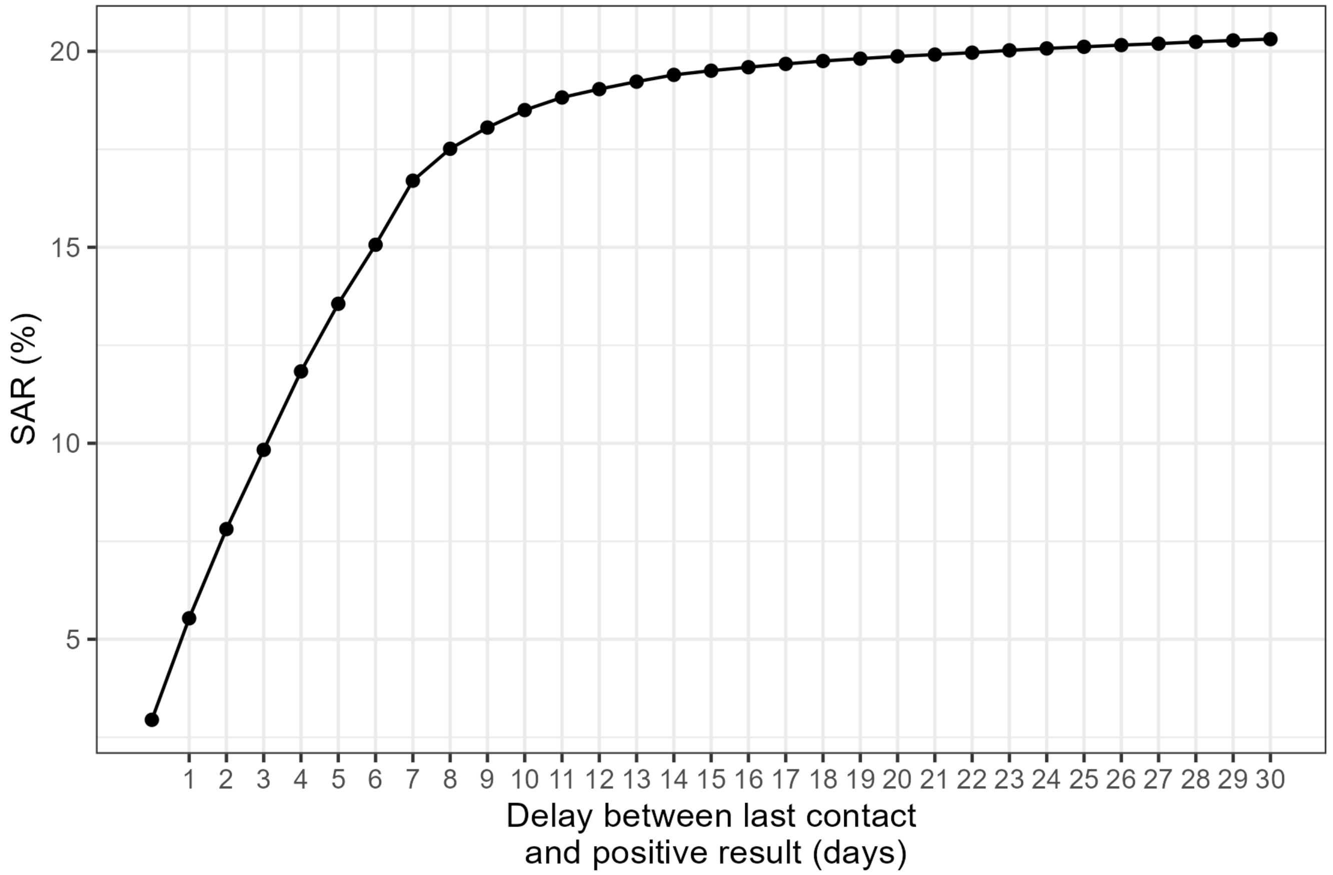

Figure S2: raw SAR as a function of the delay between test result of the index and test result of the contact.

Adjusted analysis with all immune category for index cases

		EU1	alpha	delta	omicron
	Reference	33.3*** [30.9,35.7]	31.0*** [27.8,34.1]	34.5*** [32.0,37.0]	41.0*** [37.4,44.6]
Index	Immunity: previously infected < 6months (**NVNI**)	-10.6*** [-14.7,-6.5]	-10.2*** [-15.3,-5.1]	-5.8 [-12.8,1.3]	-4.0 [-10.4,2.5]
Index	Immunity: previously infected > 6months (**NVNI**)	-7.8** [-13.3,-2.3]	-5.0 [-14.0,3.9]	-11.6*** [-14.8,-8.4]	-4.4** [-7.7,-1.1]
Index	Immunity: vaccinated < 6 months (**NVNI**)		-5.7* [-10.2,-1.2]	-5.5*** [-6.8,-4.2]	-6.4*** [-8.1,-4.7]
Index	Immunity: vaccinated > 6 months (**NVNI**)			-1.3 [-3.5,1.0]	-2.6** [-4.5,-0.7]
Index	Immunity: hybrid (**NVNI**)		19.3 [-13.3,51.9]	-8.5** [-13.7,-3.4]	-6.6*** [-9.5,-3.6]
Index	women (**men**)	-0.3 [-1.2,0.5]	0.1 [-1.4,1.5]	0.1 [-1.0,1.1]	-1.7* [-3.0,-0.4]
Index	age 0-17 (**18-64**)	-0.7 [-2.1,0.6]	-4.9*** [-6.6,-3.1]	-2.6*** [-3.8,-1.3]	-2.1* [-3.9,-0.4]
Index	age 65+ (**18-64**)	1.2 [-0.6,3.0]	3.8* [0.1,7.4]	1.5 [-0.9,4.0]	2.0 [-2.0,6.1]
Index	Obese (**not obese**)	1.1 [-0.4,2.5]	2.1 [-0.3,4.5]	0.5 [-1.5,2.4]	-0.8 [-3.0,1.4]
Index	Symptoms (**no symptoms**)	2.0 [-0.1,4.0]	7.6*** [5.1,10.0]	5.2*** [3.4,7.0]	4.5** [1.5,7.6]
index	Cough (**no cough**)	5.3*** [4.4,6.2]	5.5*** [3.9,7.1]	2.2*** [1.0,3.3]	1.8* [0.4,3.3]
index	neighbourhood poverty: slight (**wealthy**)	-0.3 [-1.6,1.0]	0.8 [-1.4,3.0]	-1.1 [-2.6,0.5]	0.2 [-1.7,2.2]
index	neighbourhood poverty: moderate (**wealthy**)	-0.9 [-2.1,0.4]	-0.9 [-3.1,1.4]	-0.7 [-2.3,0.8]	-0.4 [-2.4,1.5]
index	neighbourhood poverty: high (**wealthy**)	-0.4 [-1.5,0.7]	0.0 [-1.9,2.0]	-1.3 [-2.7,0.1]	-0.5 [-2.3,1.2]
index	vulnerable (**not vulnerable**)	-0.7 [-2.3,0.8]	-1.2 [-3.6,1.3]	0.6 [-1.5,2.6]	-2.1 [-4.9,0.8]
index	living: single house (**building**)	0.0 [-1.2,1.3]	-1.3 [-3.4,0.8]	0.0 [-1.4,1.5]	-0.9 [-2.9,1.0]
index	living: collective structure (**building**)	0.7 [-1.8,3.2]	-1.9 [-6.3,2.6]	-5.1** [-8.3,-1.8]	-0.1 [-4.3,4.1]
Index - Contact	intimate/family (**housing**)	-9.4*** [-10.3,-8.4]	-13.1*** [-14.7,-11.6]	-8.4*** [-9.6,-7.3]	-8.0*** [-9.6,-6.3]
Index - Contact	pro/school/daily (**housing**)	-10.7*** [-11.9,-9.5]	-15.3*** [-17.7,-13.0]	-10.8*** [-12.5,-9.2]	-11.5*** [-13.9,-9.0]
Contact	Immunity: previously infected < 6months (**NVNI**)	-13.3*** [-15.4,-11.3]	-26.5*** [-28.1,-24.8]	-30.7*** [-32.1,-29.4]	-32.0*** [-33.9,-30.0]
Contact	Immunity: previously infected > 6months (**NVNI**)	-17.3*** [-19.3,-15.4]	-20.6*** [-23.3,-18.0]	-14.9*** [-16.8,-12.9]	-4.4** [-7.6,-1.2]
Contact	Immunity: vaccinated < 6 months (**NVNI**)		-13.5*** [-16.0,-11.0]	-9.5*** [-10.5,-8.4]	7.0*** [5.0,8.9]
Contact	Immunity: vaccinated > 6 months (**NVNI**)			0.8 [-1.6,3.3]	13.3*** [11.2,15.4]
Contact	Immunity: hybrid (**NVNI**)		-20.1*** [-25.8,-14.3]	-21.7*** [-22.9,-20.6]	-18.2*** [-20.4,-16.1]
Contact	women (**men**)	0.2 [-0.4,0.8]	-1.0 [-2.1,0.1]	-0.2 [-1.0,0.6]	-0.4 [-1.5,0.7]
Contact	age: 0-17 (**18-64**)	-11.7*** [-12.5,-11.0]	-8.2*** [-9.5,-6.8]	-0.2 [-1.3,0.8]	-4.8*** [-6.2,-3.4]
Contact	65+ (**18-64**)	4.1*** [2.4,5.8]	1.2 [-1.7,4.1]	-1.4 [-3.2,0.3]	-6.5*** [-9.6,-3.4]
Contact	N test 90 days: 0 (**1**)	-16.3*** [-17.4,-15.1]	-11.1*** [-12.7,-9.5]	-13.3*** [-14.6,-12.0]	-17.0*** [-18.6,-15.5]
Contact	N test 90 days: 2+ (**1**)	1.3 [-1.2,3.9]	1.7 [-1.0,4.4]	3.0** [1.1,4.8]	-0.1 [-2.0,1.8]

Table S2: Estimated coefficients of the multivariable generalized estimating equation [Confidence Interval], providing the additional effect of each variable on the reference secondary attack rate (first line), for the 4 periods of dominance of the variants EU1, alpha, delta, and omicron. Estimates, p values and confidence intervals are produced by a generalized estimating equations linear regression with robust standard errors predicting a binary outcome indicating if the contact was infected by the index or not, using the index cases as cluster and an exchangeable correlation structure. The estimates are based on 42,295 index-contact relations for the EU variant, 20,311 for the alpha variant, 27,260 for the delta variant and 21,808 for the omicron variant. Estimates are adjusted for the index case gender, age, obesity, presence of symptoms, presence of cough, immunity status, neighbourhood socio-economic condition, vulnerability and type of living; the link between the index case and its contacts, and for the contact persons, their gender, age, number of tests performed the three months before the contact date with the index case, and their immunity status. The reference index case - contact relation of this multivariate analysis is the contact between two men of age below 65 living at the same place, the index being not vaccinated not infected (NVNI), not obese, living in a wealthy neighbourhood and being not a vulnerable person, living in a housing building, and the contact person being a NVNI adult men who performed one SARS-CoV-2 test during the last 3 month preceding the contact. The reference category for each categorical variable is indicated in bold in parenthesis. The left column indicates if the variable concerns the index case, the contact, or their relation. p values are indicated with *. *: 0.01<p<0.05, **:0.001<p<0.01, ***: p<0.001

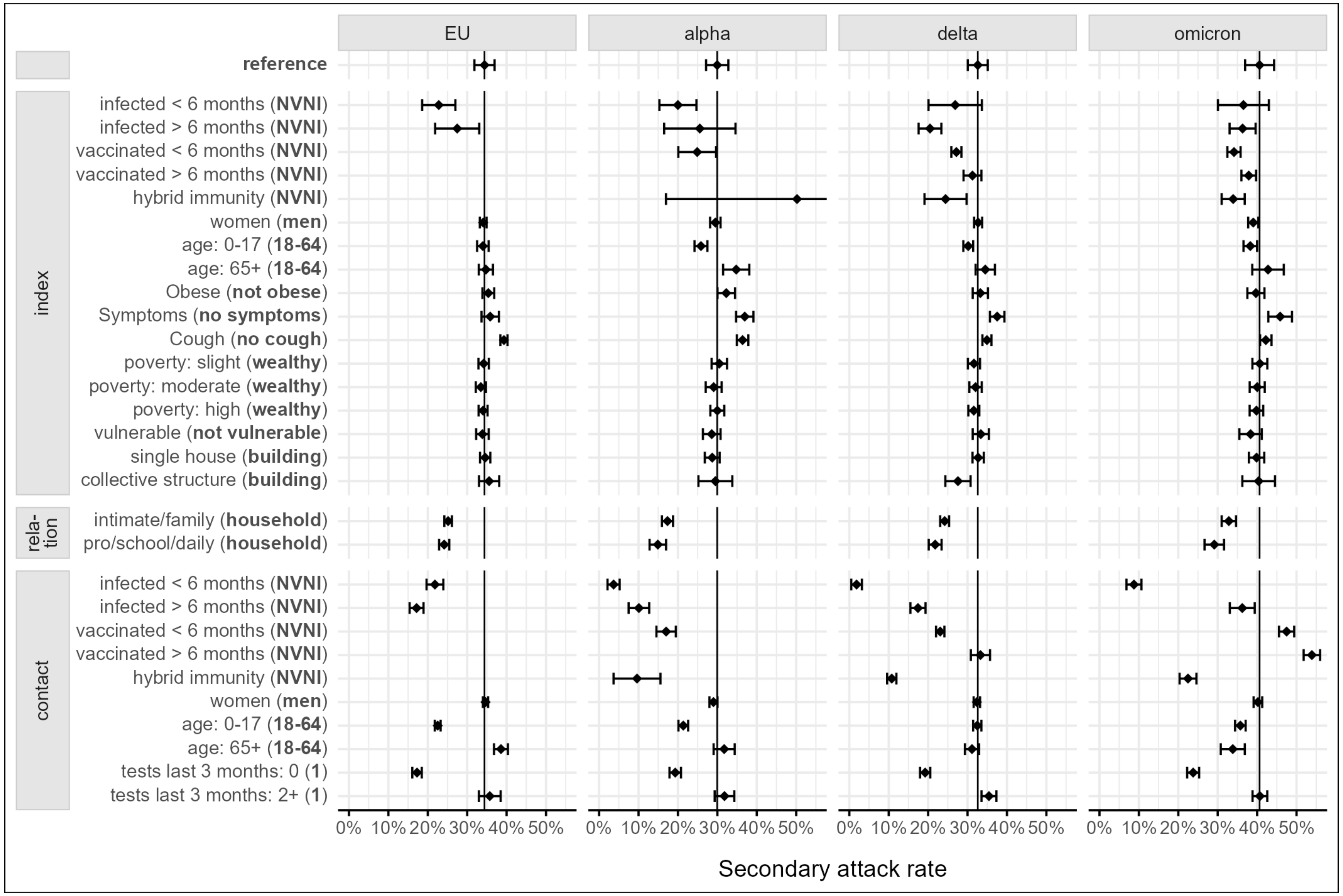

Figure S3: Estimated Adjusted Secondary Attack Rate (circle) and its 95% confidence interval (error bars) stratified per variant (EU1, alpha, delta and omicron), with the reference value indicated with a vertical line (reference of each covariate is indicated in bold in parenthesis). Estimates and confidence intervals are produced by a generalized estimating equations linear regression with robust standard errors predicting a binary outcome indicating if the contact was infected by the index or not, using the index cases as cluster and an exchangeable correlation structure. The estimates are based on 42295 index-contact relations for the EU variant, 20,311 for the alpha variant, 27,260 for the delta variant and 21,808 for the omicron variant. Estimates are adjusted for the index case gender, age, obesity, presence of symptoms, presence of cough, immunity status, neighbourhood socio-economic condition, vulnerability and type of living; the link between the index case and its contacts, and for the contact persons, their gender, age, number of tests performed the three months before the contact date with the index case, and their immunity status. The reference index case - contact relation of this multivariate analysis is the contact between two men of age below 65 living at the same place, the index being not vaccinated not infected (NVNI), not obese, living in a wealthy neighbourhood and being not a vulnerable person, living in a housing building, and the contact person being a NVNI adult men who performed one SARS-CoV-2 test during the last 3 month preceding the contact. Exact values of the estimated can be found in Table 2, and unadjusted estimates are presented in table S2.

Adjusted analysis with interaction term between contact immunity and propensity of contact to perform tests

Figure S4 and table S3 present the coefficients of the interaction terms for the multivariable analysis when including an interaction term between the number of tests performed by the contacts the last 3 months and their immunity status.

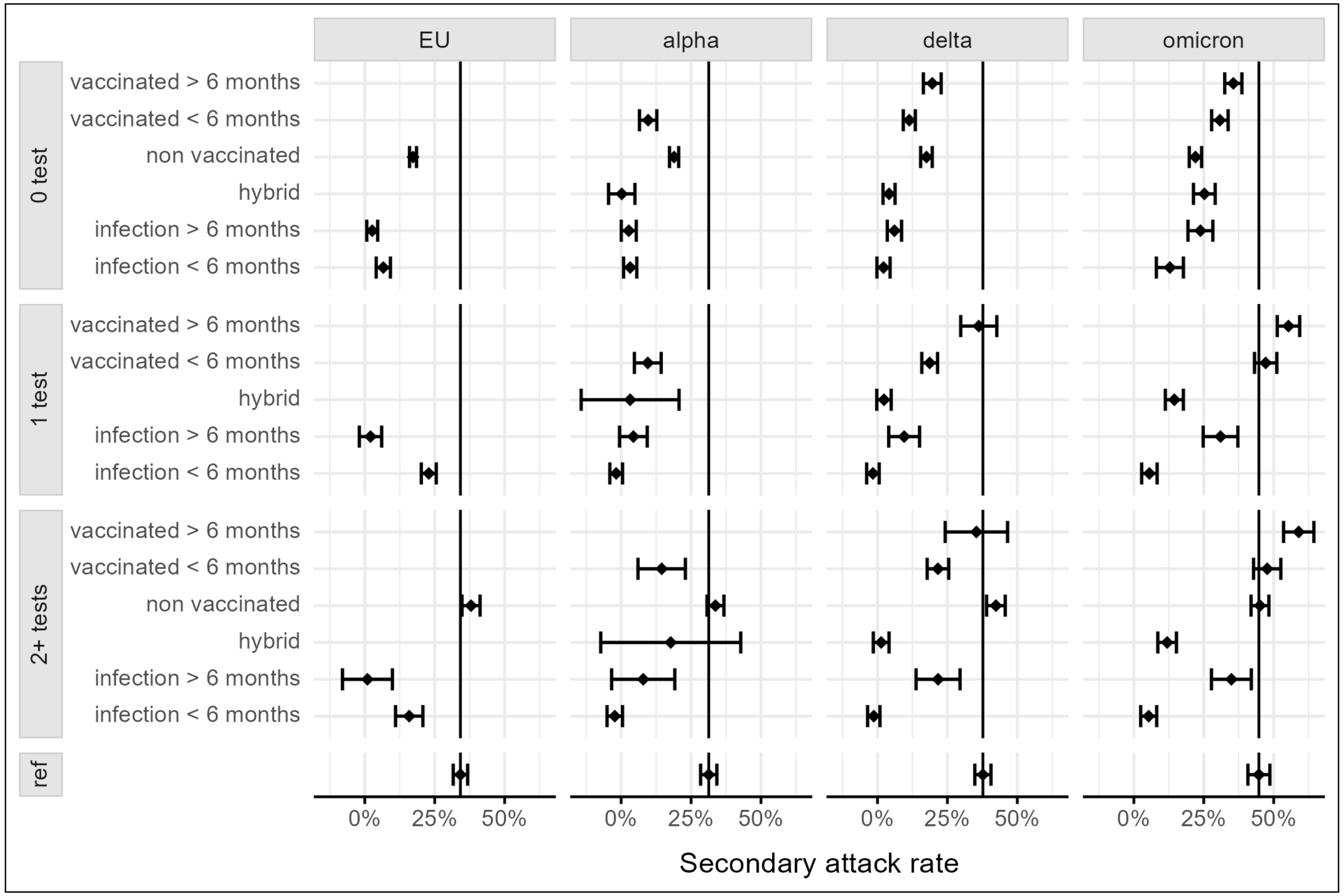

Figure S4: Adjusted secondary attack rate, displayed per immune status of the contact and number of tests performed by the contact during the 90 days preceding its last encounter with the index case. The estimates are based on 42295 index-contact relations for the EU variant, 20,311 for the alpha variant, 27,260 for the delta variant and 21,808 for the omicron variant. Estimates and confidence intervals are produced by a generalized estimating equations linear regression with robust standard errors predicting a binary outcome indicating if the contact was infected by the index or not, using the index cases as cluster and an exchangeable correlation structure. Estimates are adjusted for the index case gender, age, obesity, presence of symptoms, presence of cough, immunity status, neighbourhood socio-economic condition, vulnerability and type of living; the link between the index case and its contacts, and for the contact persons, their gender, age, number of tests performed the three months before the contact date with the index case, their immunity status, and an interaction between immunity status and number of tests performed. The reference index case - contact relation of this multivariate analysis is the contact between two men of age below 65 living at the same place, the index being not vaccinated not infected (NVNI), not obese, living in a wealthy neighbourhood and being not a vulnerable person, living in a housing building, and the contact person being a NVNI adult men who performed one SARS-CoV-2 test during the last 3 month preceding the contact.

Immune status contact	Number of tests 3 months prior to contact	EU1	alpha	delta	omicron
Infected < 6 months	0 test	-27.6*** [-30.1,-25.0]	-28.1*** [-30.5,-25.7]	-35.5*** [-37.9,-33.2]	-31.8*** [-36.7,-27.0]
Infected > 6 months	0 test	-31.5*** [-33.5,-29.6]	-28.6*** [-31.3,-25.9]	-31.6*** [-34.2,-29.1]	-20.9*** [-25.4,-16.5]
Hybrid immunity	0 test	NA* [NA,NA]	-31.1*** [-35.8,-26.4]	-33.6*** [-35.7,-31.4]	-19.5*** [-23.4,-15.6]
NVNI	0 test	-16.9*** [-18.2,-15.7]	-12.4*** [-14.0,-10.8]	-20.2*** [-22.3,-18.1]	-22.7*** [-24.9,-20.5]
Vaccinated < 6 months	0 test		-21.7*** [-24.8,-18.6]	-26.3*** [-28.5,-24.2]	-14.0*** [-16.9,-11.0]
Vaccinated > 6 months	0 test			-18.1*** [-21.3,-14.9]	-9.1*** [-12.2,-6.1]
Infected < 6 months	1 test	-11.3*** [-14.0,-8.6]	-33.1*** [-35.4,-30.8]	-39.3*** [-41.6,-37.1]	-39.2*** [-41.9,-36.4]
Infected > 6 months	1 test	-32.2*** [-36.2,-28.2]	-26.9*** [-31.9,-22.0]	-28.2*** [-33.7,-22.7]	-13.7*** [-19.9,-7.5]
hybrid	1 test		-28.1** [-45.6,-10.6]	-35.4*** [-38.0,-32.8]	-30.2*** [-33.5,-27.0]
Vaccinated < 6 months	1 test		-21.8*** [-26.6,-17.0]	-19.0*** [-21.9,-16.2]	2.4 [-1.6,6.4]
Vaccinated > 6 months	1 test			-1.5 [-7.9,5.0]	10.6*** [6.6,14.6]
Infected < 6 months	2+ tests	-18.3*** [-23.2,-13.4]	-33.6*** [-36.4,-30.8]	-39.0*** [-41.2,-36.8]	-39.4*** [-42.3,-36.6]
Infected > 6 months	2+ tests	-33.2*** [-42.2,-24.3]	-23.4*** [-34.7,-12.1]	-16.0*** [-23.9,-8.2]	-9.8** [-17.0,-2.7]
Hybrid immunity	2+ tests		-13.6 [-38.6,11.4]	-36.4*** [-39.2,-33.6]	-32.8*** [-36.1,-29.5]
NVNI	2+ tests	3.8* [0.6,7.0]	2.4 [-0.6,5.4]	4.7** [1.3,8.0]	0.4 [-2.8,3.6]
Vaccinated < 6 months	2+ tests		-16.8*** [-25.3,-8.3]	-16.1*** [-19.9,-12.2]	3.0 [-1.9,7.8]
Vaccinated > 6 months	2+ tests			-2.3 [-13.5,8.8]	14.2*** [8.8,19.7]
	ref	34.2*** [31.6,36.8]	31.3*** [28.4,34.2]	37.7*** [34.8,40.6]	44.7*** [40.8,48.6]

Table S3: Coefficients [Confidence Interval] of the interaction terms between the immune status of the contact and the number of tests performed by the contacts 3 months prior to the date of the interaction between contact and index, for the adjusted multivariable model. These coefficients provide the additional effect on the reference secondary attack rate (last line), for the 4 periods of dominance of the variants EU1, alpha, delta, and omicron. The estimates are based on 42,295 index-contact relations for the EU variant, 20,311 for the alpha variant, 27,260 for the delta variant and 21,808 for the omicron variant. Estimates and confidence intervals are produced by a generalized estimating equations linear regression with robust standard errors predicting a binary outcome indicating if the contact was infected by the index or not, using the index cases as cluster and an exchangeable correlation structure. Estimates are adjusted for the index case gender, age, obesity, presence of symptoms, presence of cough, immunity status, neighbourhood socio-economic condition, vulnerability and type of living; the link between the index case and its contacts, and for the contact persons, their gender, age, number of tests performed the three months before the contact date with the index case, their immunity status, and an interaction between immunity status and number of tests performed. The reference index case - contact relation of this multivariate analysis is the contact between two men of age below 65 living at the same place, the index being not vaccinated not infected (NVNI), not obese, living in a wealthy neighbourhood and being not a vulnerable person, living in a housing building, and the contact person being a NVNI adult men who performed one SARS-CoV-2 test during the last 3 month preceding the contact. The left column indicates if the variable concerns the index case, the contact, or their relation. p values are indicated with *. *: 0.01<p<0.05, **:0.001<p<0.01, ***: p<0.001. NVNI : non vaccinated never infected.

Univariable and multivariable analysis

Some of the variables had a different effect when performing a univariable analysis (see supplementary table S4) or when adjusting for all co-variate. The main differences concerned the effect of symptoms during the alpha wave (+12.3pp in the univariable model and +7.0pp in the adjusted model) and in a lesser extend during the EU1 wave (4.8 pp in the univariable model and 2pp in the adjusted model). This difference was caused by the adjustment for the cough and for demographic variables.
The Immune status of the contact had different effects between the univariable and adjusted model. The effect of being vaccinated less than 6 months ago for the contact was greater in the adjusted analysis, especially for the omicron wave.
The SAR reduction associated with the fact of being previously infected was larger in the adjusted analysis than in the univariable analysis, especially during the EU1, delta and omicron. These differences were mainly due to the adjustment for the propensity of the contacts to perform tests, measured by the number of COVID-19 tests performed during the last 3 months. In a sensitivity analysis including an interaction term between the immune status of the contact and the number of tests performed the last 3 months (see supplementary table S3), we observed a highly significant interaction between these two variables. This interaction effect is mainly driven by the fact that non-vaccinated and vaccinated more than 6 months ago individuals who didn’t do any tests in the last 3 months had low SAR compared to those with a higher propensity to test. In contrast, previously infected individuals had very low SAR irrespective of their number of previous tests.

On the opposite, the protection of being previously infected for the index case was lowered in the adjusted analysis when compared with the univariable analysis. The adjustment for symptoms and cough explained 2pp of this difference, and the adjustment immune status of the contact explained the rest of this difference.

	variable	EU1	alpha	delta	omicron
Index	Immunity: previously infected < 6months (**NVNI**)	-10.7*** [-14.5,-6.8]	-18.7*** [-22.9,-14.6]	-13.8*** [-20.7,-6.9]	-11.5*** [-17.8,-5.3]
Index	Immunity: previously infected > 6months (**NVNI**)	-12.7*** [-17.9,-7.4]	-10.6* [-19.3,-2.0]	-12.8*** [-15.7,-9.9]	-7.1*** [-10.4,-3.8]
Index	Immunity: vaccinated > 6 months (**NVNI**)		-4.7* [-9.0,-0.4]	-6.5*** [-7.7,-5.3]	-5.4*** [-7.1,-3.8]
Index	Immunity: vaccinated < 6 months (**NVNI**)			-1.0 [-3.1,1.1]	-0.4 [-2.2,1.4]
Index	Immunity: hybrid (**NVNI**)		11.6 [-29.6,52.8]	-10.6*** [-15.9,-5.3]	-8.2*** [-11.0,-5.4]
Index	women (**men**)	-0.6 [-1.5,0.2]	0.4 [-1.1,2.0]	-0.0 [-1.1,1.0]	-1.9** [-3.1,-0.7]
Index	age 0-17 (**18-64**)	-2.9*** [-4.3,-1.5]	-8.2*** [-9.9,-6.4]	-0.3 [-1.4,0.8]	1.6* [0.2,3.1]
Index	age 65+ (**18-64**)	4.2*** [2.4,6.0]	0.2 [-3.4,3.9]	-0.3 [-2.8,2.1]	1.5 [-2.4,5.3]
Index	Obese (**not obese**)	1.8* [0.2,3.3]	3.6** [1.0,6.1]	0.6 [-1.4,2.5]	-1.1 [-3.3,1.0]
Index	Symptoms (**no symptoms**)	4.9*** [2.8,6.9]	12.3*** [10.1,14.4]	5.4*** [3.7,7.1]	5.1*** [2.4,7.9]
index	Cough (**no cough**)	5.9*** [5.0,6.8]	8.1*** [6.6,9.6]	2.9*** [1.9,4.0]	1.6* [0.3,3.0]
index	Immunity: cured (**not vaccinated**)	-11.0*** [-14.2,-7.8]	-16.3*** [-20.4,-12.3]	-12.8*** [-15.4,-10.2]	-5.5*** [-8.4,-2.5]
index	Immunity: vaccinated > 6 months (**not vaccinated**)			-1.8 [-3.7,0.2]	-0.8 [-2.4,0.8]
index	Immunity: vaccinated < 6 months (**not vaccinated**)		-5.2* [-9.4,-1.1]	-6.6*** [-7.7,-5.4]	-5.0*** [-6.4,-3.5]
index	neighbourhood poverty: slight (**wealthy**)	-0.3 [-1.7,1.0]	1.1 [-1.2,3.4]	-1.1 [-2.6,0.5]	0.2 [-1.6,2.0]
index	neighbourhood poverty: moderate (**wealthy**)	-1.0 [-2.2,0.3]	-0.5 [-2.7,1.8]	-0.7 [-2.2,0.8]	-0.7 [-2.5,1.1]
index	neighbourhood poverty: high (**wealthy**)	-0.6 [-1.7,0.5]	0.7 [-1.2,2.7]	-0.9 [-2.2,0.4]	-0.9 [-2.5,0.7]
index	vulnerable (**not vulnerable**)	-1.1 [-2.7,0.5]	-0.5 [-3.0,2.0]	0.5 [-1.5,2.5]	-3.3* [-5.9,-0.6]
index	living: single house (**building**)	0.8 [-0.4,2.0]	-1.2 [-3.3,0.9]	-0.3 [-1.7,1.0]	0.1 [-1.7,1.9]
index	living: collective structure (**building**)	0.5 [-2.1,3.1]	-4.7* [-9.2,-0.1]	-6.0*** [-8.9,-3.0]	-1.3 [-5.1,2.5]
Index - Contact	intimate/family (**housing**)	-8.7*** [-9.6,-7.8]	-12.9*** [-14.4,-11.3]	-9.0*** [-10.1,-7.9]	-6.5*** [-8.3,-4.8]
Index - Contact	pro/school/daily (**housing**)	-10.4*** [-11.6,-9.3]	-16.3*** [-18.6,-14.0]	-12.2*** [-13.6,-10.7]	-10.7*** [-13.5,-7.9]
Contact	Immunity: previously infected < 6months (**NVNI**)	1.3 [-0.6,3.1]	-21.5*** [-22.9,-20.2]	-22.7*** [-23.7,-21.7]	-21.4*** [-23.0,-19.8]
Contact	Immunity: previously infected > 6months (**NVNI**)	-17.0*** [-18.6,-15.4]	-20.0*** [-22.4,-17.5]	-15.5*** [-17.4,-13.5]	-1.3 [-4.5,1.8]
Contact	Immunity: vaccinated < 6 months (**NVNI**)		-10.5*** [-12.8,-8.2]	-10.6*** [-11.6,-9.5]	9.0*** [7.1,10.9]
Contact	Immunity: vaccinated > 6 months (**NVNI**)		-14.9* [-17.5,-12.3]	-1.1 [-3.5,1.4]	16.7*** [14.7,18.7]
Contact	Immunity: hybrid (**NVNI**)		-17.5*** [-22.9,-12.2]	-21.0*** [-22.0,-20.0]	-10.5*** [-12.3,-8.7]
Contact	women (**men**)	0.5 [-0.1,1.2]	-1.2 [-2.3,0.0]	-0.1 [-0.9,0.7]	0.3 [-0.7,1.4]
Contact	age: 0-17 (**18-64**)	-12.1*** [-12.8,-11.4]	-6.8*** [-8.1,-5.5]	2.4*** [1.5,3.4]	-9.5*** [-10.6,-8.4]
Contact	65+ (**18-64**)	3.5*** [1.8,5.2]	-4.0** [-6.9,-1.2]	-2.5** [-4.3,-0.7]	-7.8*** [-10.8,-4.9]
Contact	N test 30days: 0 (**1**)	-16.6*** [-17.7,-15.5]	-10.8*** [-12.4,-9.2]	-13.5*** [-14.8,-12.2]	-15.3*** [-17.0,-13.7]
Contact	N test 30days: 2+ (**1**)	1.5 [-1.1,4.1]	1.0 [-1.9,3.9]	2.4* [0.1,4.6]	-4.0** [-6.9,-1.1]

Table S4: Coefficients [Confidence Interval] of the univariable generalized estimating equations linear regression with robust standard errors predicting a binary outcome indicating if the contact was infected by the index or not, using the index cases as cluster and an exchangeable correlation structure. The estimates are based on 42295 index-contact relations for the EU variant, 20,311 for the alpha variant, 27,260 for the delta variant and 21,808 for the omicron variant.. Each coefficient yields the additional effect of the variable on the secondary attack rate for the 4 periods of dominance of the variants EU1, alpha, delta, and omicron. The reference category for each categorical variable is indicated in bold in parenthesis. The left column indicates if the variable concerns the index case, the contact, or their relation. p values are indicated with *. *: 0.01<p<0.05, **:0.001<p<0.01, ***: p<0.001

Adjusted analysis stratified per gender

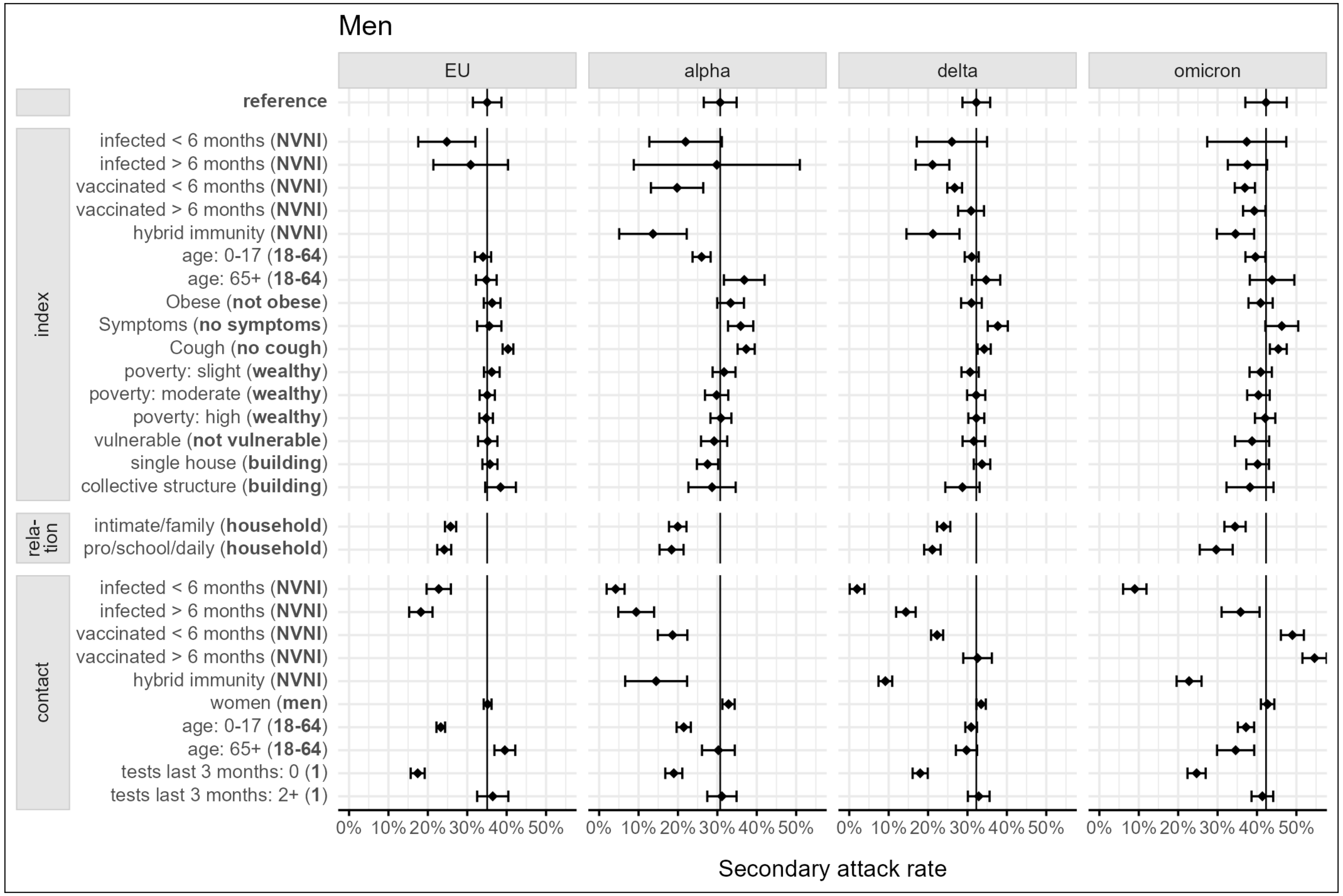

Figure S5a: Estimated Adjusted Secondary Attack Rate (diamond) and its 95% confidence interval (error bars) stratified per variant (EU1, alpha, delta and omicron) considering only men, with the reference value indicated with a vertical line (reference of each covariate is indicated in bold in parenthesis). Estimates and confidence intervals are produced by a generalized estimating equations linear regression with robust standard errors predicting a binary outcome indicating if the contact was infected by the index or not, using the index cases as cluster and an exchangeable correlation structure. The estimates are based on 19,756 index-contact relations for the EU variant, 9,337 for the alpha variant, 12,830 for the delta variant and 9,760 for the omicron variant. Estimates are adjusted for the index case gender, age, obesity, presence of symptoms, presence of cough, immunity status, neighbourhood socio-economic condition, vulnerability and type of living; the link between the index case and its contacts, and for the contact persons, their gender, age, number of tests performed the three months before the contact date with the index case, and their immunity status. The reference index case - contact relation of this multivariate analysis is the contact between two men of age below 65 living at the same place, the index being not vaccinated not infected (NVNI), not obese, living in a wealthy neighbourhood and being not a vulnerable person, living in a housing building, and the contact person being a NVNI adult men who performed one SARS-CoV-2 test during the last 3 month preceding the contact. Exact values of the estimated can be found in Table 2, and unadjusted estimates are presented in table S2.

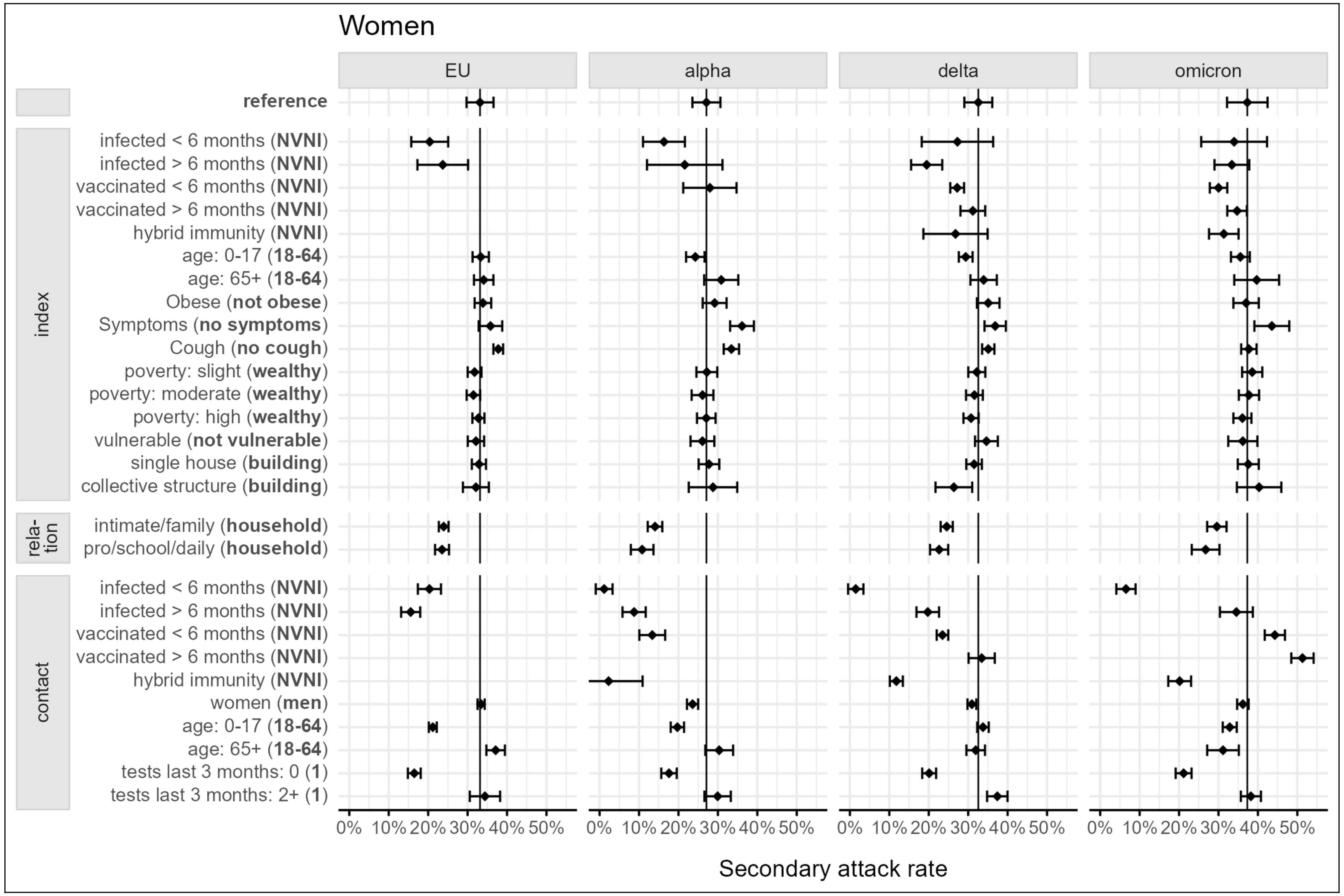

Figure S5b: Estimated Adjusted Secondary Attack Rate (diamond) and its 95% confidence interval (error bars) stratified per variant (EU1, alpha, delta and omicron) considering only women, with the reference value indicated with a vertical line (reference of each covariate is indicated in bold in parenthesis). Estimates and confidence intervals are produced by a generalized estimating equations linear regression with robust standard errors predicting a binary outcome indicating if the contact was infected by the index or not, using the index cases as cluster and an exchangeable correlation structure. The estimates are based on 22,520 index-contact relations for the EU variant, 10,938 for the alpha variant, 14,325 for the delta variant and 11,955 for the omicron variant. Estimates are adjusted for the index case gender, age, obesity, presence of symptoms, presence of cough, immunity status, neighbourhood socio-economic condition, vulnerability and type of living; the link between the index case and its contacts, and for the contact persons, their gender, age, number of tests performed the three months before the contact date with the index case, and their immunity status. The reference index case - contact relation of this multivariate analysis is the contact between two men of age below 65 living at the same place, the index being not vaccinated not infected (NVNI), not obese, living in a wealthy neighbourhood and being not a vulnerable person, living in a housing building, and the contact person being a NVNI adult men who performed one SARS-CoV-2 test during the last 3 month preceding the contact. Exact values of the estimated can be found in Table 2, and unadjusted estimates are presented in table S2.

Sensitivity analysis

Adjusted analysis using complete case analysis

Multivariable analysis

We present here the result of the multivariable analysis but considering only the complete case dataset, which is of 48'468 lines. Figure S6 and table S5 present the result of this analysis. Figure S7 and table S6 present the coefficients of the interaction term for the same analysis when considering an interaction term between the number of tests performed by the contacts the last 3 months and their immunity status.

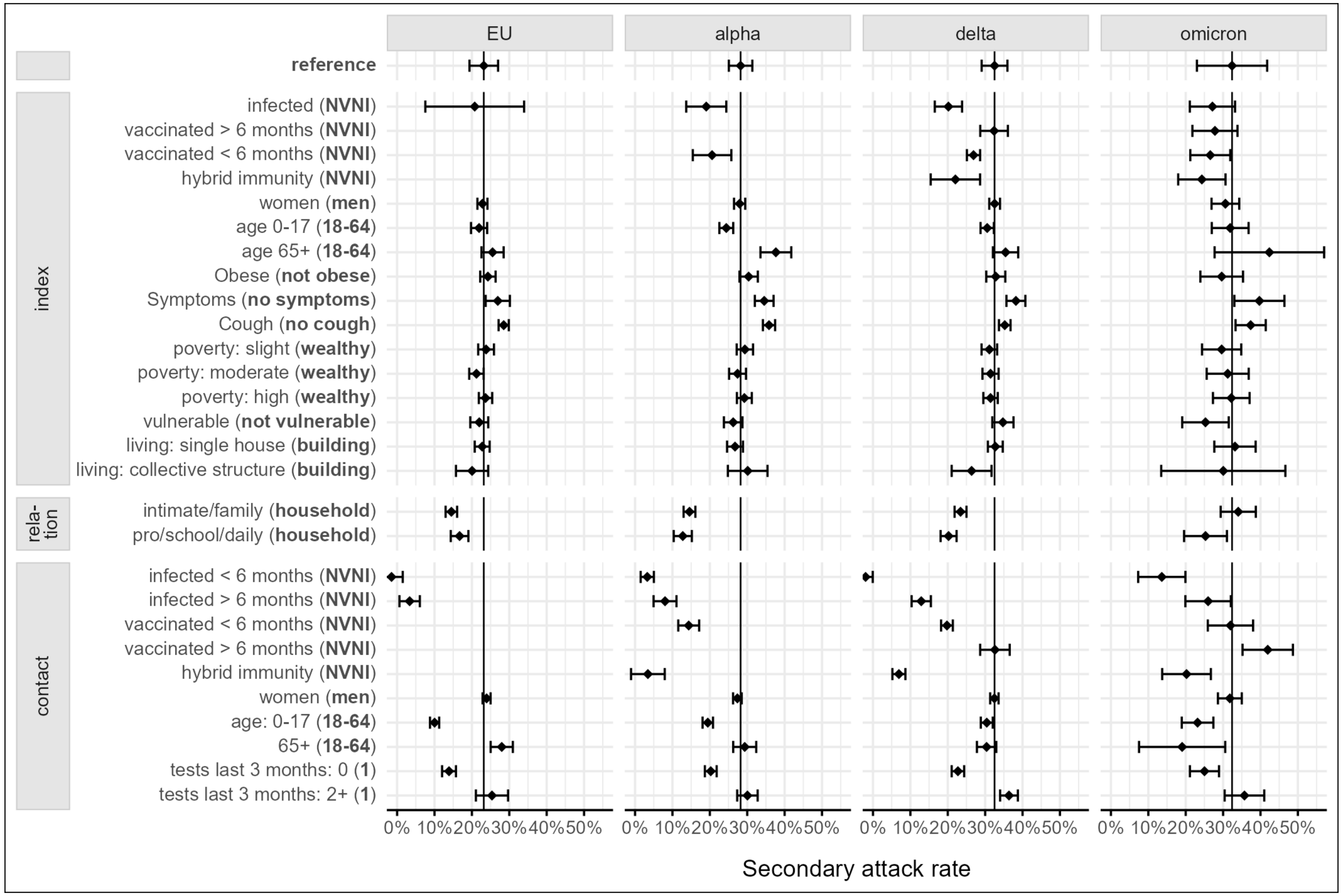

Figure S6: Adjusted Secondary Attack Rate (SAR) stratified per variant (EU1, alpha, delta and omicron), with the reference value indicated with a vertical line (reference of each covariate is indicated in bold in parenthesis), for the complete case analysis (no missing value for any covariate). Estimates and confidence intervals are produced by a generalized estimating equations linear regression with robust standard errors predicting a binary outcome indicating if the contact was infected by the index or not, using the index cases as cluster and an exchangeable correlation structure. The estimates are based on 14,790 index-contact relations for the EU variant, 16,356 for the alpha variant, 14,801 for the delta variant and 2,521 for the omicron variant. Estimates are adjusted for the index case gender, age, obesity, presence of symptoms, presence of cough, immunity status, neighbourhood socio-economic condition, vulnerability and type of living; the link between the index case and its contacts, and for the contact persons, their gender, age, number of tests performed the three months before the contact date with the index case, their immunity status, and an interaction between immunity status and number of tests performed. The reference index case - contact relation of this multivariate analysis is the contact between two men of age below 65 living at the same place, the index being not vaccinated not infected (NVNI), not obese, living in a wealthy neighbourhood and being not a vulnerable person, living in a housing building, and the contact person being a NVNI adult men who performed one SARS-CoV-2 test during the last 3 month preceding the contact.

		EU1	alpha	delta	omicron
	Reference	23.2*** [19.4,27.0]	28.2*** [25.1,31.4]	32.5*** [29.1,36.0]	32.4*** [23.0,41.8]
index	Immunity: previously infected (**NVNI**)	-2.4 [-15.6,10.8]	-9.2*** [-14.5,-3.8]	-12.3*** [-16.0,-8.7]	-5.2 [-11.3,0.8]
index	Immunity: vaccinated < 6 months (**NVNI**)			-0.1 [-3.8,3.6]	-4.6 [-10.6,1.5]
index	Immunity: vaccinated > 6 months (**NVNI**)		-7.6** [-12.8,-2.5]	-5.6*** [-7.4,-3.9]	-5.8* [-11.2,-0.5]
index	Hybrid immunity (**NVNI**)		19.4 [-12.6,51.5]	-10.5** [-17.1,-3.9]	-8.1* [-14.4,-1.7]
index	women (**men**)	-0.4 [-1.7,1.0]	-0.3 [-1.8,1.2]	0.0 [-1.4,1.5]	-1.8 [-5.5,1.9]
index	age 0-17 (**18-64**)	-1.3 [-3.4,0.9]	-3.8*** [-5.7,-2.0]	-2.0* [-3.7,-0.2]	-0.5 [-5.4,4.4]
index	age 65+ (**18-64**)	2.4 [-0.6,5.3]	9.4*** [5.3,13.6]	2.9 [-0.4,6.3]	10.0 [-4.7,24.6]
index	Obese (**not obese**)	1.1 [-1.0,3.2]	2.2 [-0.3,4.6]	0.3 [-2.2,2.9]	-2.8 [-8.5,2.9]
index	Symptoms (**no symptoms**)	3.7* [0.5,7.0]	6.3*** [3.8,8.8]	5.7*** [3.2,8.2]	7.3* [0.6,14.0]
index	Cough (**no cough**)	5.3*** [4.0,6.7]	7.6*** [6.0,9.2]	2.7*** [1.2,4.3]	5.0* [0.9,9.0]
index	neighbourhood poverty: slight (**wealthy**)	0.6 [-1.5,2.7]	1.1 [-1.0,3.3]	-1.4 [-3.5,0.7]	-2.8 [-8.0,2.5]
index	neighbourhood poverty: moderate (**wealthy**)	-2.0* [-3.9,-0.0]	-0.8 [-3.1,1.4]	-1.1 [-3.2,1.1]	-1.2 [-6.8,4.5]
index	neighbourhood poverty: high (**wealthy**)	0.5 [-1.3,2.3]	1.0 [-1.0,3.0]	-1.1 [-3.0,0.9]	-0.2 [-5.1,4.7]
index	vulnerable (**not vulnerable**)	-1.2 [-3.6,1.2]	-2.0 [-4.5,0.5]	2.2 [-0.6,5.1]	-7.1* [-13.3,-0.9]
index	living: single house (**building**)	-0.4 [-2.4,1.6]	-1.5 [-3.6,0.6]	0.2 [-1.8,2.2]	0.8 [-4.7,6.3]
index	living: collective structure (**building**)	-3.1 [-7.4,1.2]	1.9 [-3.4,7.2]	-6.1* [-11.5,-0.8]	-2.3 [-18.9,14.3]
Index - Contact	intimate/family (**housing**)	-8.7*** [-10.2,-7.1]	-13.7*** [-15.2,-12.1]	-9.1*** [-10.7,-7.5]	1.7 [-3.0,6.4]
Index - Contact	pro/school/daily (**housing**)	-6.5*** [-8.8,-4.1]	-15.5*** [-17.9,-13.0]	-12.3*** [-14.4,-10.2]	-7.1* [-12.8,-1.4]
Contact	Immunity: previously infected < 6months (**NVNI**)	-24.7*** [-27.7,-21.7]	-25.0*** [-26.7,-23.2]	-34.5*** [-36.4,-32.6]	-18.8*** [-25.1,-12.5]
contact	Immunity: previously infected > 6months (**NVNI**)	-19.8*** [-22.6,-17.1]	-20.2*** [-23.3,-17.1]	-19.6*** [-22.2,-17.0]	-6.4* [-12.5,-0.3]
contact	Immunity: vaccinated < 6 months (**NVNI**)		-13.9*** [-16.7,-11.1]	-12.7*** [-14.3,-11.2]	-0.4 [-6.5,5.6]
contact	Immunity: vaccinated > 6 months (**NVNI**)			0.1 [-3.8,4.1]	9.5** [2.8,16.3]
contact	Immunity: hybrid (**NVNI**)		-24.8*** [-29.3,-20.3]	-25.6*** [-27.3,-23.8]	-12.2*** [-18.7,-5.7]
contact	women (**men**)	0.8 [-0.3,1.8]	-0.9 [-2.0,0.3]	-0.0 [-1.2,1.1]	-0.6 [-3.8,2.6]
contact	age: 0-17 (**18-64**)	-13.2*** [-14.4,-11.9]	-8.8*** [-10.2,-7.4]	-2.1* [-3.7,-0.5]	-9.2*** [-13.4,-5.0]
contact	65+ (**18-64**)	4.8** [1.8,7.8]	1.1 [-2.0,4.2]	-2.1 [-4.7,0.5]	-13.3* [-24.9,-1.8]
contact	Number of tests last 90 days: 0 (**1**)	-9.3*** [-11.2,-7.4]	-8.0*** [-9.6,-6.4]	-9.8*** [-11.5,-8.1]	-7.4*** [-11.3,-3.5]
contact	Number of tests last 90 days: 2+ (**1**)	2.2 [-2.1,6.5]	1.8 [-0.9,4.6]	3.9** [1.5,6.2]	3.3 [-2.0,8.6]

table S5: Estimated coefficients of the multivariable generalized estimating equation [Confidence Interval] when considering only complete case (i.e. data with no missing covariates), providing the additional effect of each variable on the reference secondary attack rate (first line), for the 4 periods of dominance of the variants EU1, alpha, delta, and omicron. Estimates, p values and confidence intervals are produced by a generalized estimating equations linear regression with robust standard errors predicting a binary outcome indicating if the contact was infected by the index or not, using the index cases as cluster and an exchangeable correlation structure. The estimates are based on 14,790 index-contact relations for the EU variant, 16,356 for the alpha variant, 14,801 for the delta variant and 2,521 for the omicron variant. Estimates are adjusted for the index case gender, age, obesity, presence of symptoms, presence of cough, immunity status, neighbourhood socio-economic condition, vulnerability and type of living; the link between the index case and its contacts, and for the contact persons, their gender, age, number of tests performed the three months before the contact date with the index case, and their immunity status. The reference index case - contact relation of this multivariate analysis is the contact between two men of age below 65 living at the same place, the index being not vaccinated not infected (NVNI), not obese, living in a wealthy neighbourhood and being not a vulnerable person, living in a housing building, and the contact person being a NVNI adult men who performed one SARS-CoV-2 test during the last 3 month preceding the contact. The reference category for each categorical variable is indicated in bold in parenthesis. The left column indicates if the variable concerns the index case, the contact, or their relation. p values are indicated with *. *: $0.01<p<0.05$, **: $0.001<p<0.01$, ***: $p<0.001$

Multivariable analysis with interaction term

We present the results of the multivariable analysis for the complete case analysis considering an interaction term between the propensity to perform test and the immunity of the contact in Figure S7 and table S6.

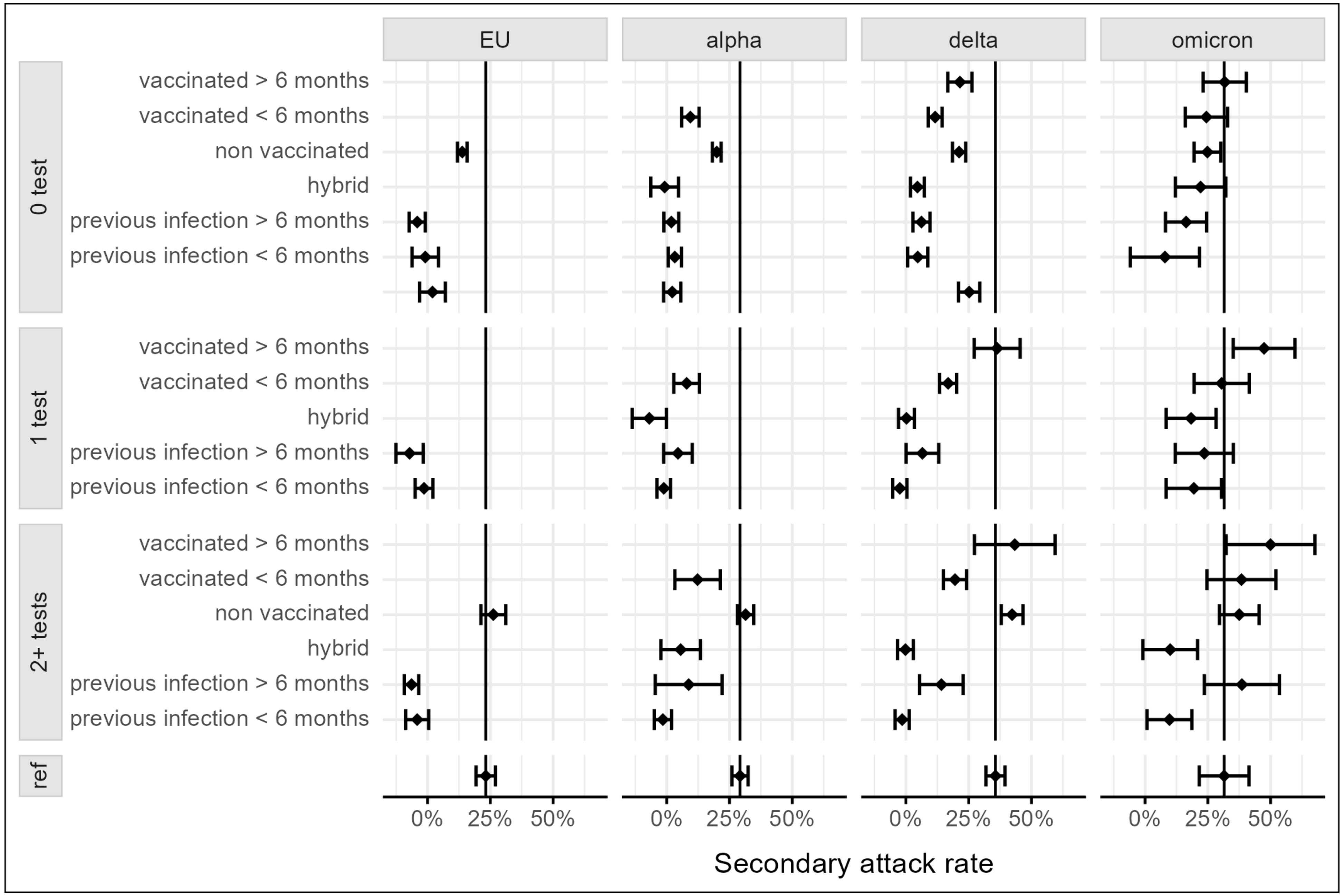

Figure S7: Adjusted secondary attack rate, displayed per immune status of the contact and number of tests performed by the contact during the 90 days preceding its last encounter with the index case, for the complete case (i.e. data with no missing covariates). Estimates and confidence intervals are produced by a generalized estimating equations linear regression with robust standard errors predicting a binary outcome indicating if the contact was infected by the index or not, using the index cases as cluster and an exchangeable correlation structure. The estimates are based on 14,790 index-contact relations for the EU variant, 16,356 for the alpha variant, 14,801 for the delta variant and 2,521 for the omicron variant. Estimates are adjusted for the index case gender, age, obesity, presence of symptoms, presence of cough, immunity status, neighbourhood socio-economic condition, vulnerability and type of living; the link between the index case and its contacts, and for the contact persons, their gender, age, number of tests performed the three months before the contact date with the index case, their immunity status, and an interaction between immunity status and number of tests performed. The reference index case - contact relation of this multivariate analysis is the contact between two men of age below 65 living at the same place, the index being not vaccinated not infected (NVNI), not obese, living in a wealthy neighbourhood and being not a vulnerable person, living in a housing building, and the contact person being a NVNI adult men who performed one SARS-CoV-2 test during the last 3 month preceding the contact.

Immunity status	Number of test last 90 days	EU	alpha	delta	omicron
Infected < 6 months	0 test	-24.1*** [-29.3,-18.8]	-26.0*** [-28.6,-23.3]	-31.1*** [-35.2,-27.1]	-24.2*** [-37.7,-10.8]
Infected > 6 months	0 test	-27.3*** [-30.5,-24.1]	-27.4*** [-30.3,-24.4]	-29.4*** [-32.8,-26.0]	-14.9*** [-23.1,-6.7]
Hybrid immunity	0 test		-30.0*** [-35.5,-24.5]	-31.1*** [-33.8,-28.3]	-9.2 [-19.2,0.9]
NVNI	0 test	-9.4*** [-11.3,-7.5]	-9.3*** [-11.0,-7.5]	-14.5*** [-17.1,-11.9]	-6.6* [-12.0,-1.3]
Vaccinated < 6 months	0 test		-19.8*** [-23.3,-16.3]	-24.0*** [-26.8,-21.3]	-7.0 [-15.5,1.4]
Vaccinated > 6 months	0 test			-14.2*** [-19.0,-9.3]	0.3 [-8.3,8.9]
Infected < 6 months	1 test	-24.6*** [-28.1,-21.1]	-30.4*** [-33.1,-27.7]	-38.2*** [-41.0,-35.3]	-12.3* [-23.3,-1.4]
Infected > 6 months	1 test	-30.4*** [-35.8,-24.9]	-24.7*** [-30.4,-19.0]	-29.1*** [-35.6,-22.6]	-7.7 [-19.3,3.8]
hybrid	1 test		-36.1*** [-42.9,-29.3]	-35.4*** [-38.6,-32.2]	-13.2** [-23.1,-3.2]
Vaccinated < 6 months	1 test		-21.3*** [-26.4,-16.1]	-18.8*** [-22.2,-15.5]	-0.9 [-11.9,10.0]
Vaccinated > 6 months	1 test			0.7 [-8.5,9.8]	16.0* [3.7,28.3]
Infected < 6 months	2+ tests	-27.3*** [-31.9,-22.7]	-30.7*** [-34.2,-27.3]	-37.2*** [-40.0,-34.4]	-22.1*** [-31.0,-13.2]
Infected > 6 months	2+ tests	-29.6*** [-32.5,-26.7]	-20.5** [-33.7,-7.2]	-21.5*** [-30.2,-12.8]	7.3 [-7.6,22.3]
Hybrid immunity	2+ tests		-23.7*** [-31.5,-15.8]	-35.9*** [-39.0,-32.8]	-21.3*** [-32.2,-10.4]
NVNI	2+ tests	3.0 [-1.9,8.0]	2.2 [-1.1,5.5]	6.6** [2.3,11.0]	6.0 [-1.9,13.9]
Vaccinated < 6 months	2+ tests		-16.9*** [-26.0,-7.9]	-16.1*** [-20.7,-11.5]	6.9 [-6.9,20.6]
Vaccinated > 6 months	2+ tests			7.7 [-8.4,23.7]	18.4* [0.7,36.1]
	reference	23.2*** [19.3,27.0]	29.2*** [26.0,32.4]	35.6*** [31.8,39.4]	31.4*** [21.5,41.3]

Table S6: Coefficients [Confidence Interval] of the interaction terms between the immune status of the contact and the number of tests performed by the contacts 3 months prior to the date of the interaction between contact and index, for the adjusted multivariable model when considering complete case (i.e. data with no missing caovraites). These coefficients provide the additional effect on the reference secondary attack rate (last line), for the 4 periods of dominance of the variants EU1, alpha, delta, and omicron. The estimates are based on 14,790 index-contact relations for the EU variant, 16,356 for the alpha variant, 14,801 for the delta variant and 2,521 for the omicron variant. Estimates and confidence intervals are produced by a generalized estimating equations linear regression with robust standard errors predicting a binary outcome indicating if the contact was infected by the index or not, using the index cases as cluster and an exchangeable correlation structure. Estimates are adjusted for the index case gender, age, obesity, presence of symptoms, presence of cough, immunity status, neighbourhood socio-economic condition, vulnerability and type of living; the link between the index case and its contacts, and for the contact persons, their gender, age, number of tests performed the three months before the contact date with the index case, their immunity status, and an interaction between immunity status and number of tests performed. The reference index case - contact relation of this multivariate analysis is the contact between two men of age below 65 living at the same place, the index being not vaccinated not infected (NVNI), not obese, living in a wealthy neighbourhood and being not a vulnerable person, living in a housing building, and the contact person being a NVNI adult men who performed one SARS-CoV-2 test during the last 3 month preceding the contact. The left column indicates if the variable concerns the index case, the contact, or their relation. p values are indicated with *. *: $0.01<p<0.05$, **:$0.001<p<0.01$, ***: $p<0.001$. NVNI : non vaccinated never infected.

Adjusted analysis using the number of tests performed in the 180 days before the index-contact interaction as a proxy for testing

In order to test the robustness of the number of tests performed the 3 months (90 days) preceding the last index-contact interaction as a proxy of the contact to get tested, we propose a sensitivity analysis using instead the number of tests performed by the contact the last 180 days instead of the last 90 days, discretized in 4 categories: 0, 1, 2, and 3+. Descriptive statistics of this new variable can be found in table S7, and the result of the multivariable analysis in Figure S8 and table S8. The results of the adjusted analysis with the interaction between immune status and the propensity to test is displayed Figure S9 and table S9.

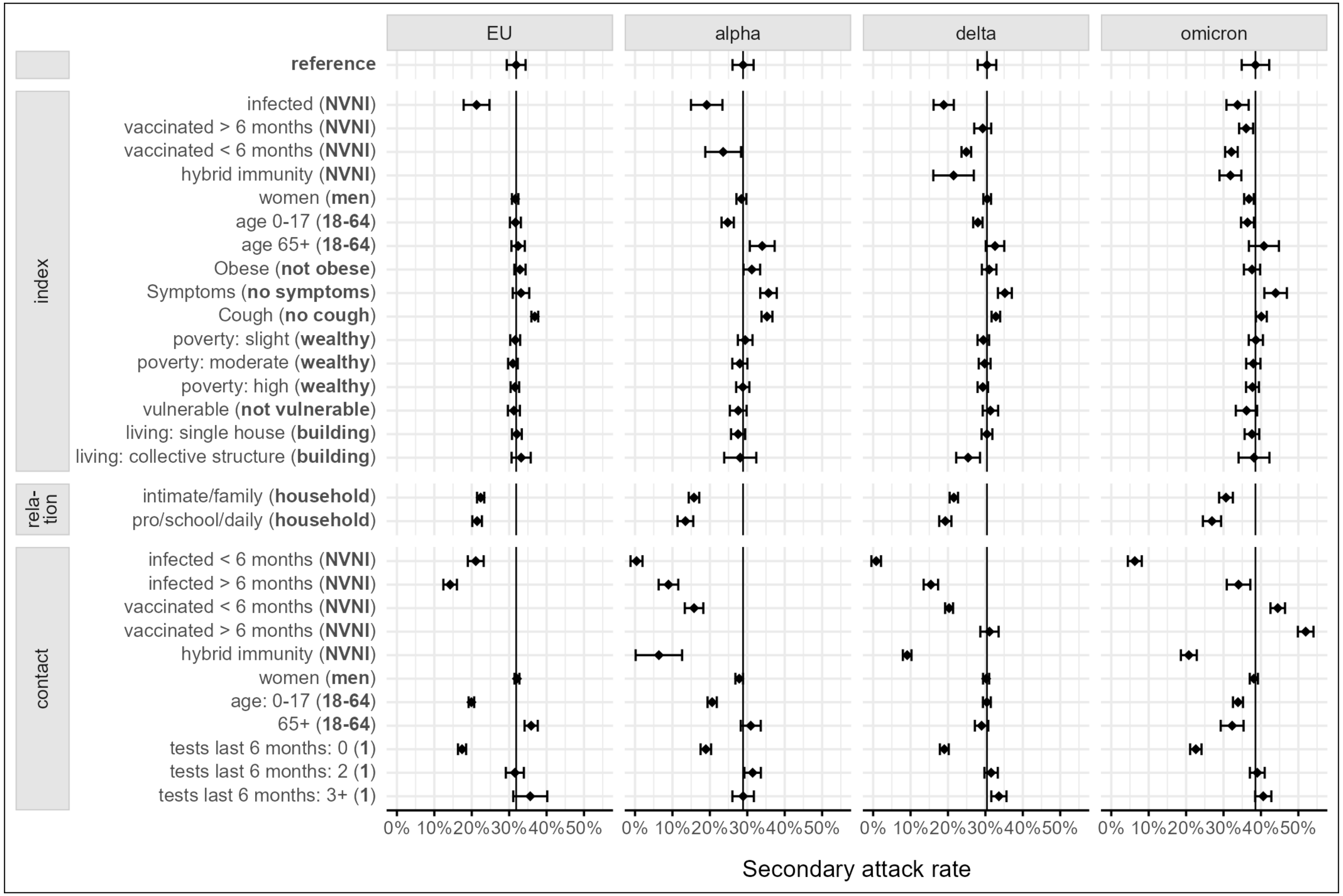

Figure S8: Adjusted Secondary Attack Rate (SAR) stratified per variant (EU1, alpha, delta and omicron), with the reference value indicated with a vertical line (reference of each covariate is indicated in bold in parenthesis). Estimates and confidence intervals are produced by a generalized estimating equations linear regression with robust standard errors predicting a binary outcome indicating if the contact was infected by the index or not, using the index cases as cluster and an exchangeable correlation structure. The estimates are based on 42,295 index-contact relations for the EU variant, 20,311 for the alpha variant, 27,260 for the delta variant and 21,808 for the omicron variant. Estimates are adjusted for the index case gender, age, obesity, presence of symptoms, presence of cough, immunity status, neighbourhood socio-economic condition, vulnerability and type of living; the link between the index case and its contacts, and for the contact persons, their gender, age, number of tests performed the six months before the contact date with the index case, and their immunity status. The reference index case - contact relation of this multivariate analysis is the contact between two men of age below 65 living at the same place, the index being not vaccinated not infected (NVNI), not obese, living in a wealthy neighbourhood and being not a vulnerable person, living in a housing building, and the contact person being a NVNI adult men who performed one SARS-CoV-2 test during the last 6 month preceding the contact.

	Overall	EU1	Alpha	Delta	Omicron
Number of tests performed the last 6 months (%)					
1	24680 (22.1)	5008 (24.7)	6171 (22.6)	8244 (19.5)	5257 (24.1)
0	73097 (65.5)	12689 (62.5)	16630 (61.0)	32202 (76.1)	11576 (53.1)
2	8511 (7.6)	1745 (8.6)	2578 (9.5)	1490 (3.5)	2698 (12.4)
3+	5386 (4.8)	869 (4.3)	1881 (6.9)	359 (0.8)	2277 (10.4)

Table S7: descriptive statistics of the Number of tests performed by the contact during the last 6 months preceding their last encounter with the index case, stratified by variant.

		EU1	alpha	delta	omicron
	Reference	31.9*** [29.4,34.4]	28.9*** [26.1,31.7]	30.4*** [27.9,32.9]	38.5*** [34.9,42.2]
index	Immunity: previously infected (**NVNI**)	-10.6*** [-14.0,-7.1]	-9.7*** [-13.9,-5.5]	-11.6*** [-14.3,-8.9]	-4.8** [-7.8,-1.8]
index	Immunity: vaccinated < 6 months (**NVNI**)			-1.1 [-3.4,1.1]	-2.5** [-4.4,-0.6]
index	Immunity: vaccinated > 6 months (**NVNI**)		-5.3* [-10.1,-0.5]	-5.5*** [-6.8,-4.2]	-6.4*** [-8.1,-4.7]
index	Hybrid immunity (**NVNI**)		18.2 [-16.6,52.9]	-8.9** [-14.3,-3.5]	-6.7*** [-9.6,-3.8]
index	women (**men**)	-0.3 [-1.1,0.6]	-0.4 [-1.8,0.9]	0.1 [-1.0,1.1]	-1.7** [-3.0,-0.4]
index	age 0-17 (**18-64**)	-0.2 [-1.7,1.3]	-4.1*** [-5.7,-2.5]	-2.4*** [-3.7,-1.2]	-2.1* [-3.9,-0.4]
index	age 65+ (**18-64**)	0.5 [-1.3,2.3]	5.1** [1.8,8.4]	2.1 [-0.3,4.6]	2.3 [-1.8,6.3]
index	Obese (**not obese**)	1.0 [-0.5,2.5]	2.3* [0.1,4.6]	0.6 [-1.4,2.5]	-0.9 [-3.1,1.3]
index	Symptoms (**no symptoms**)	1.3 [-0.9,3.5]	6.8*** [4.6,9.0]	4.8*** [2.9,6.6]	5.4*** [2.4,8.4]
index	Cough (**no cough**)	5.0*** [4.1,5.9]	6.4*** [4.9,7.8]	2.4*** [1.2,3.5]	1.6* [0.1,3.0]
index	neighbourhood poverty: slight (**wealthy**)	-0.3 [-1.6,1.1]	0.6 [-1.4,2.5]	-1.0 [-2.5,0.6]	0.1 [-1.8,2.0]
index	neighbourhood poverty: moderate (**wealthy**)	-0.9 [-2.2,0.4]	-0.9 [-2.9,1.2]	-0.6 [-2.2,1.0]	-0.6 [-2.5,1.3]
index	neighbourhood poverty: high (**wealthy**)	-0.3 [-1.5,0.8]	-0.1 [-1.8,1.7]	-1.1 [-2.5,0.3]	-0.8 [-2.5,0.9]
index	vulnerable (**not vulnerable**)	-0.6 [-2.2,1.0]	-1.3 [-3.5,0.9]	0.9 [-1.1,3.0]	-2.4 [-5.3,0.4]
index	living: single house (**building**)	0.2 [-1.1,1.5]	-1.3 [-3.2,0.6]	-0.0 [-1.4,1.4]	-0.9 [-2.9,1.0]
index	living: collective structure (**building**)	1.3 [-1.2,3.9]	-0.8 [-5.0,3.5]	-5.0** [-8.2,-1.9]	-0.4 [-4.5,3.7]
Index - Contact	intimate/family (**housing**)	-9.5*** [-10.5,-8.5]	-13.1*** [-14.5,-11.7]	-8.9*** [-10.0,-7.7]	-7.9*** [-9.7,-6.0]
Index - Contact	pro/school/daily (**housing**)	-10.4*** [-11.7,-9.2]	-15.4*** [-17.5,-13.3]	-11.1*** [-12.8,-9.5]	-11.6*** [-14.0,-9.2]
Contact	Immunity: previously infected < 6months (**NVNI**)	-10.8*** [-12.9,-8.7]	-28.5*** [-30.1,-26.9]	-29.6*** [-30.9,-28.3]	-32.2*** [-34.1,-30.4]
contact	Immunity: previously infected > 6months (**NVNI**)	-17.6*** [-19.5,-15.8]	-19.9*** [-22.6,-17.3]	-15.0*** [-16.9,-13.1]	-4.5** [-7.7,-1.4]
contact	Immunity: vaccinated < 6 months (**NVNI**)		-13.1*** [-15.6,-10.6]	-10.1*** [-11.2,-9.1]	6.0*** [4.0,7.9]
contact	Immunity: vaccinated > 6 months (**NVNI**)			0.7 [-1.8,3.1]	13.4*** [11.3,15.5]
contact	Immunity: hybrid (**NVNI**)		-22.5*** [-28.7,-16.3]	-21.3*** [-22.5,-20.2]	-17.8*** [-19.9,-15.7]
contact	women (**men**)	0.2 [-0.5,0.9]	-1.0* [-2.0,-0.0]	-0.2 [-1.0,0.6]	-0.4 [-1.5,0.7]
contact	age: 0-17 (**18-64**)	-12.0*** [-12.7,-11.2]	-8.3*** [-9.5,-7.0]	-0.0 [-1.1,1.0]	-4.7*** [-6.0,-3.3]
contact	65+ (**18-64**)	4.0*** [2.2,5.8]	2.1 [-0.6,4.7]	-1.4 [-3.2,0.3]	-6.2*** [-9.3,-3.2]
contact	Number of tests last 180 days: 0 (**1**)	17.4*** [16.3,18.5]	18.9*** [17.5,20.3]	19.0*** [17.8,20.2]	22.6*** [21.1,24.1]
contact	Number of tests last 180 days: 2 (**1**)	31.5 [29.1,33.9]	31.4* [29.2,33.6]	31.5 [29.7,33.3]	39.0 [37.0,41.0]
contact	Number of tests last 180 days: 3+ (**1**)	35.6 [31.1,40.2]	28.9 [26.0,31.8]	33.6** [31.6,35.6]	40.6 [38.5,42.8]

table S8: Estimated coefficients of the multivariable generalized estimating equation [Confidence Interval], providing the additional effect of each variable on the reference secondary attack rate (first line), for the 4 periods of dominance of the variants EU1, alpha, delta, and omicron. Estimates, p values and confidence intervals are produced by a generalized estimating equations linear regression with robust standard errors predicting a binary outcome indicating if the contact was infected by the index or not, using the index cases as cluster and an exchangeable correlation structure. The estimates are based on 42,295 index-contact relations for the EU variant, 20,311 for the alpha variant, 27,260 for the delta variant and 21,808 for the omicron variant. Estimates are adjusted for the index case gender, age, obesity, presence of symptoms, presence of cough, immunity status, neighbourhood socio-economic condition, vulnerability and type of living; the link between the index case and its contacts, and for the contact persons, their gender, age, number of tests performed the six months before the contact date with the

index case, and their immunity status. The reference index case - contact relation of this multivariate analysis is the contact between two men of age below 65 living at the same place, the index being not vaccinated not infected (NVNI), not obese, living in a wealthy neighbourhood and being not a vulnerable person, living in a housing building, and the contact person being a NVNI adult men who performed one SARS-CoV-2 test during the last 6 month preceding the contact. The reference category for each categorical variable is indicated in bold in parenthesis. The left column indicates if the variable concerns the index case, the contact, or their relation. p values are indicated with *. *: 0.01<p<0.05, **:0.001<p<0.01, ***: p<0.001

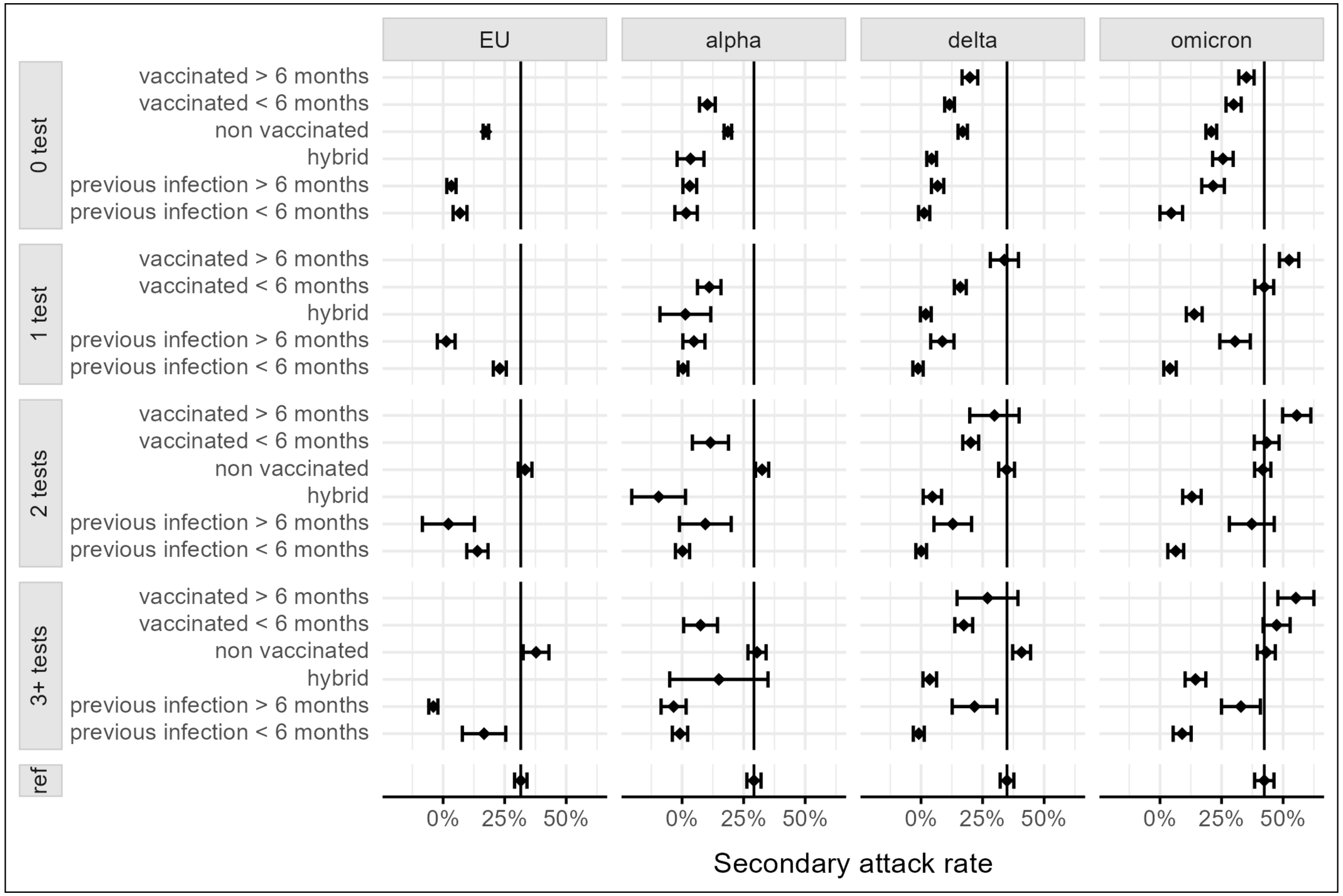

Figure S9: Adjusted secondary attack rate, displayed per immune status of the contact and number of tests performed by the contact during the 180 days preceding its last encounter with the index case, for the complete case (i.e. data with no missing covariates). Estimates and confidence intervals are produced by a generalized estimating equations linear regression with robust standard errors predicting a binary outcome indicating if the contact was infected by the index or not, using the index cases as cluster and an exchangeable correlation structure. The estimates are based on 42,295 index-contact relations for the EU variant, 20,311 for the alpha variant, 27,260 for the delta variant and 21,808 for the omicron variant. Estimates are adjusted for the index case gender, age, obesity, presence of symptoms, presence of cough, immunity status, neighbourhood socio-economic condition, vulnerability and type of living; the link between the index case and its contacts, and for the contact persons, their gender, age, number of tests performed the six months before the contact date with the index case, their immunity status, and an interaction between immunity status and number of tests performed. The reference index case - contact relation of this multivariate analysis is the contact between two men of age below 65 living at the same place, the index being not vaccinated not infected (NVNI), not obese, living in a wealthy neighbourhood and being not a vulnerable person, living in a housing building, and the contact person being a NVNI adult men who performed one SARS-CoV-2 test during the last 6 month preceding the contact.

Immunity status	Number of test the last 6 months	EU	alpha	delta	omicron
	reference	31.5*** [29.0,34.1]	29.2*** [26.3,32.1]	34.9*** [32.1,37.7]	42.4*** [38.4,46.3]
Infected < 6 months	0 test	-24.7*** [-27.5,-21.8]	-27.5*** [-32.1,-23.0]	-33.7*** [-35.9,-31.4]	-37.7*** [-42.3,-33.2]
Infected > 6 months	0 test	-28.2*** [-30.1,-26.3]	-26.2*** [-29.0,-23.4]	-28.2*** [-30.7,-25.7]	-20.9*** [-25.5,-16.3]
Hybrid immunity	0 test		-25.8*** [-31.2,-20.3]	-30.6*** [-32.6,-28.6]	-16.8*** [-21.0,-12.7]
NVNI	0 test	-14.2*** [-15.3,-13.1]	-10.6*** [-12.1,-9.1]	-18.0*** [-19.9,-16.1]	-21.5*** [-23.7,-19.3]
Vaccinated < 6 months	0 test		-18.9*** [-22.1,-15.7]	-23.3*** [-25.3,-21.4]	-12.5*** [-15.5,-9.4]
Vaccinated > 6 months	0 test			-15.1*** [-18.3,-11.9]	-7.3*** [-10.4,-4.1]
Infected < 6 months	1 test	-8.5*** [-11.1,-5.9]	-28.8*** [-30.8,-26.8]	-36.2*** [-38.3,-34.1]	-38.3*** [-40.8,-35.8]
Infected > 6 months	1 test	-30.3*** [-33.9,-26.7]	-24.5*** [-29.0,-20.1]	-26.2*** [-31.0,-21.5]	-11.9*** [-18.1,-5.7]
hybrid	1 test		-27.8*** [-38.1,-17.5]	-33.0*** [-35.2,-30.8]	-28.5*** [-31.7,-25.2]
Vaccinated < 6 months	1 test		-18.2*** [-23.0,-13.4]	-19.0*** [-21.4,-16.5]	-0.0 [-3.9,3.8]
Vaccinated > 6 months	1 test			-1.1 [-6.8,4.7]	10.1*** [6.1,14.0]
Infected < 6 months	2 tests	-17.6*** [-21.9,-13.3]	-29.0*** [-31.8,-26.1]	-34.9*** [-37.0,-32.7]	-35.9*** [-39.1,-32.7]
Infected > 6 months	2 tests	-29.4*** [-40.0,-18.8]	-19.9*** [-30.3,-9.5]	-22.0*** [-29.7,-14.4]	-5.1 [-14.2,4.0]
Hybrid immunity	2 tests		-38.7*** [-49.6,-27.8]	-30.3*** [-34.1,-26.6]	-29.4*** [-33.2,-25.7]
NVNI	2 tests	1.8 [-1.0,4.5]	3.3* [0.6,6.0]	-0.2 [-3.4,3.1]	-0.6 [-3.9,2.7]
Vaccinated < 6 months	2 tests		-17.7*** [-25.0,-10.4]	-14.7*** [-18.0,-11.5]	1.0 [-4.1,6.0]
Vaccinated > 6 months	2 tests			-5.1 [-15.1,4.9]	13.2*** [7.4,18.9]
Infected < 6 months	3+ tests	-14.9*** [-23.7,-6.1]	-30.0*** [-33.1,-26.9]	-35.8*** [-38.1,-33.6]	-33.3*** [-36.9,-29.6]
Infected > 6 months	3+ tests	-35.5*** [-37.4,-33.7]	-32.9*** [-38.0,-27.7]	-13.2** [-22.3,-4.1]	-9.5* [-17.4,-1.6]
Hybrid immunity	3+ tests		-14.3 [-34.2,5.7]	-31.4*** [-34.2,-28.6]	-28.0*** [-32.2,-23.8]
NVNI	3+ tests	6.2* [1.0,11.5]	1.2 [-2.4,4.9]	5.9** [2.3,9.6]	0.8 [-2.8,4.5]
Vaccinated < 6 months	3+ tests		-21.7*** [-28.6,-14.8]	-17.6*** [-21.2,-13.9]	5.0 [-0.5,10.5]
Vaccinated > 6 months	3+ tests			-7.9 [-20.3,4.4]	12.8*** [5.5,20.2]

Table S9: Coefficients [Confidence Interval] of the interaction terms between the immune status of the contact and the number of tests performed by the contacts 6 months prior to the date of the interaction between contact and index, for the adjusted multivariable model when considering complete case (i.e. data with no missing caovraites). These coefficients provide the additional effect on the reference secondary attack rate (last line), for the 4 periods of dominance of the variants EU1, alpha, delta, and omicron. Estimates and confidence intervals are produced by a generalized estimating equations linear regression with robust standard errors predicting a binary outcome indicating if the contact was infected by the index or not, using the index cases as cluster and an exchangeable correlation structure. The estimates are based on 42,295 index-contact relations for the EU variant, 20,311 for the alpha variant, 27,260 for the delta variant and 21,808 for the omicron variant. Estimates are adjusted for the index case gender, age, obesity, presence of symptoms, presence of cough, immunity status, neighbourhood socio-economic condition, vulnerability and type of living; the link between the index case and its contacts, and for the contact persons, their gender, age, number of tests performed the six months before the contact date with the index case, their immunity status, and an interaction between immunity status and number of tests performed. The reference index case - contact relation of this multivariate analysis is the contact between two men of age below 65 living at the same place, the index being not vaccinated not infected (NVNI), not obese, living in a wealthy neighbourhood and being not a vulnerable person, living in a housing building, and the contact person being a NVNI adult men who performed one SARS-CoV-2 test during the last 6 month preceding the contact. The left column indicates if the variable concerns the index case, the contact, or their relation. p values are indicated with *. *: 0.01<p<0.05, **:0.001<p<0.01, ***: p<0.001. NVNI : non vaccinated never infected.

Adjusted analysis using periods with variant dominance of more than 90%

We test here the sensibility of our analysis to the definitions of the periods of variant dominance. In the main text, the periods of variant dominance were defined as the periods where one VoC is >50% of the variant detected. We propose here the same analysis using the periods with >90% (see figure below). The results of the multivariable analysis are provided in Figure S10 and table S10.

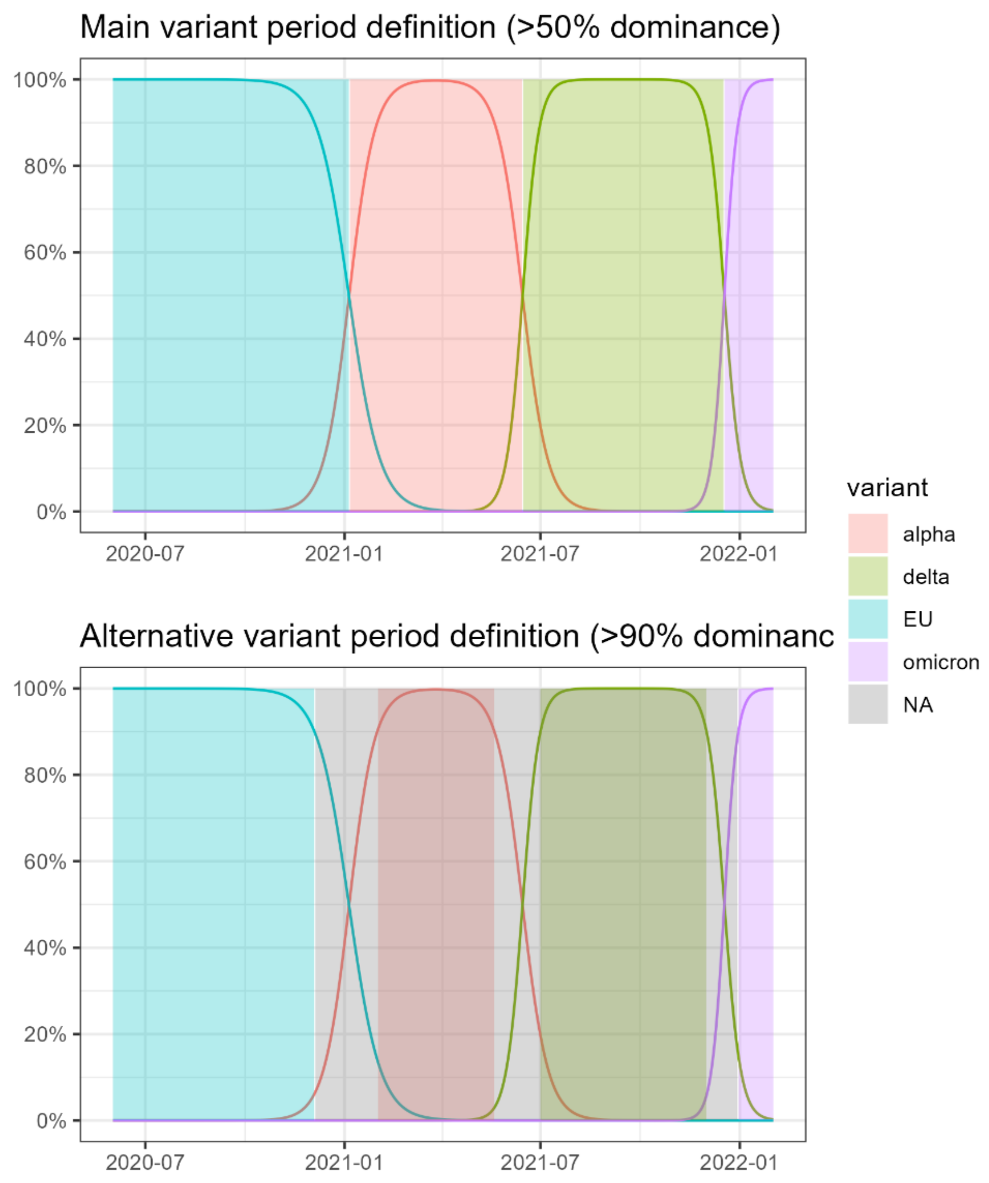

Initial periods were:

VoC	EU	alpha	delta	Omicron
Start	01-06-2020	06-01-2021	15-06-2021	18-12-2021
end	05-01-2021	14-06-2021	17-12-2021	01-02-2022

The periods are in this sensitivity analysis:

VoC	EU	alpha	delta	Omicron
Start	01-06-2020	01-02-2021	01-07-2021	31-12-2021
end	04-12-2020	19-05-2021	01-12-2021	01-02-2022

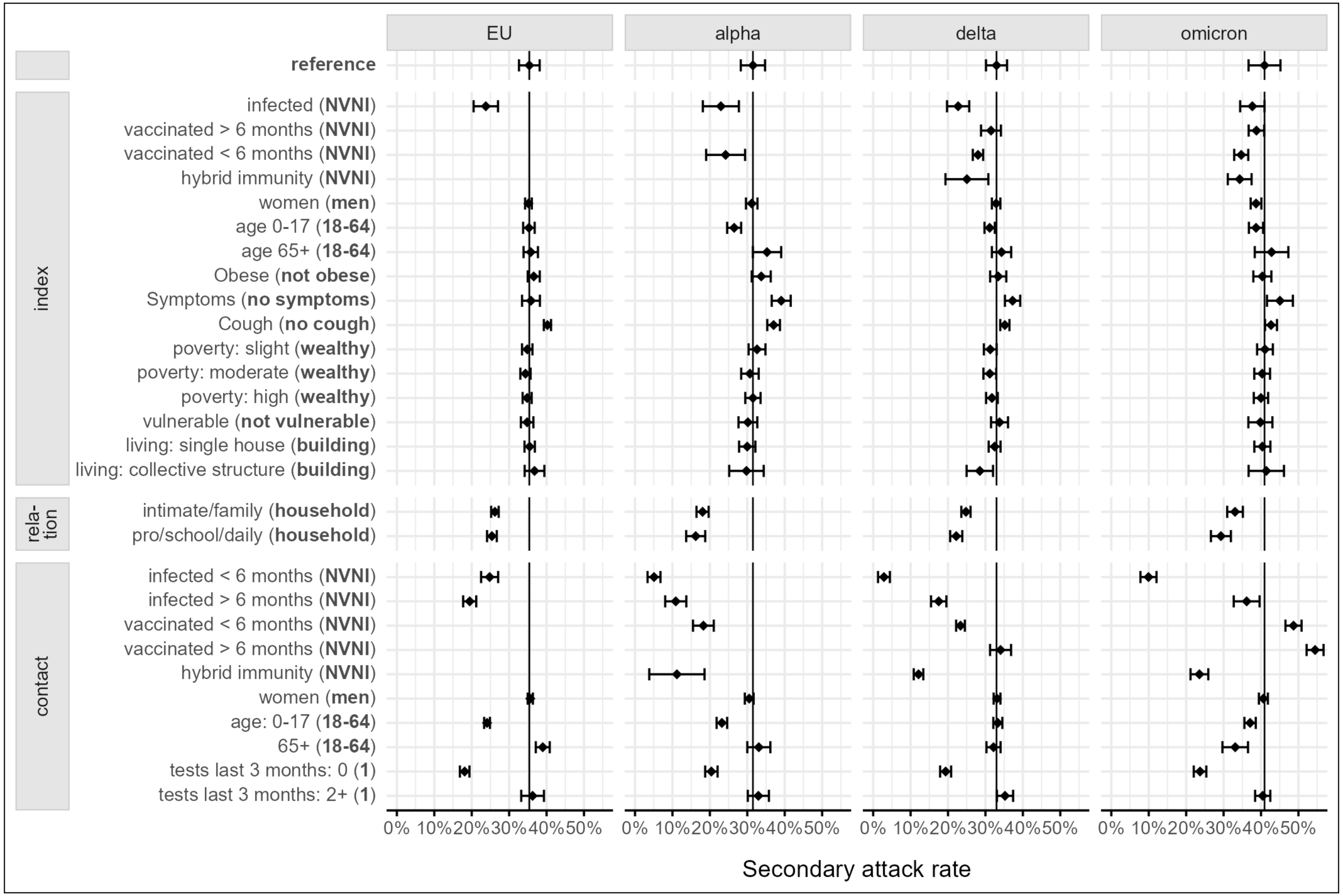

Figure S10: Adjusted Secondary Attack Rate (SAR) stratified per variant when considering period of predominance above 90% of variant share (EU1, alpha, delta and omicron), the reference value indicated with a vertical line (reference of each covariate is indicated in bold in parenthesis). Estimates and confidence intervals are produced by a generalized estimating equations linear regression with robust standard errors predicting a binary outcome indicating if the contact was infected by the index or not, using the index cases as cluster and an exchangeable correlation structure. The estimates are based on 38,148 index-contact relations for the EU variant, 15,662 for the alpha variant, 21,360 for the delta variant and 17,692 for the omicron variant. Estimates are adjusted for the index case gender, age, obesity, presence of symptoms, presence of cough, immunity status, neighbourhood socio-economic condition, vulnerability and type of living; the link between the index case and its contacts, and for the contact persons, their gender, age, number of tests performed the six months before the contact date with the index case, and their immunity status. The reference index case - contact relation of this multivariate analysis is the contact between two men of age below 65 living at the same place, the index being not vaccinated not infected (NVNI), not obese, living in a wealthy neighbourhood and being not a vulnerable person, living in a housing building, and the contact person being a NVNI adult men who performed one SARS-CoV-2 test during the last 6 month preceding the contact.

		EU1	alpha	delta	omicron
	Reference	35.4*** [32.6,38.2]	31.5*** [28.3,34.7]	33.0*** [30.1,35.8]	40.9*** [36.7,45.2]
index	Immunity: previously infected (**NVNI**)	-11.6*** [-14.9,-8.4]	-8.6*** [-13.4,-3.7]	-10.3*** [-13.2,-7.3]	-3.3 [-6.5,0.0]
index	Immunity: vaccinated < 6 months (**NVNI**)			-1.5 [-4.1,1.2]	-2.2* [-4.2,-0.1]
index	Immunity: vaccinated > 6 months (**NVNI**)		-7.3** [-12.5,-2.1]	-5.0*** [-6.3,-3.6]	-6.2*** [-8.1,-4.3]
index	Hybrid immunity (**NVNI**)		25.2 [-9.2,59.6]	-7.9** [-13.6,-2.2]	-6.6*** [-9.8,-3.5]
index	women (**men**)	-0.2 [-1.1,0.7]	-0.3 [-1.9,1.2]	-0.1 [-1.2,1.0]	-2.3** [-3.7,-0.8]
index	age 0-17 (**18-64**)	-0.1 [-1.7,1.4]	-5.0*** [-6.9,-3.1]	-1.8** [-3.2,-0.4]	-2.3* [-4.2,-0.4]
index	age 65+ (**18-64**)	0.4 [-1.5,2.3]	3.8 [-0.0,7.5]	1.3 [-1.2,3.9]	1.9 [-2.6,6.4]
index	Obese (**not obese**)	1.2 [-0.4,2.8]	2.2 [-0.3,4.8]	0.5 [-1.7,2.6]	-0.6 [-3.0,1.8]
index	Symptoms (**no symptoms**)	0.4 [-1.9,2.8]	7.6*** [5.0,10.1]	4.3*** [2.2,6.3]	4.1* [0.7,7.6]
index	Cough (**no cough**)	4.8*** [3.9,5.8]	5.5*** [3.8,7.2]	2.2*** [1.0,3.5]	1.7* [0.1,3.3]
index	neighbourhood poverty: slight (**wealthy**)	-0.6 [-2.0,0.8]	1.1 [-1.2,3.3]	-1.7 [-3.4,0.0]	0.1 [-2.0,2.2]
index	neighbourhood poverty: moderate (**wealthy**)	-1.0 [-2.4,0.3]	-0.8 [-3.1,1.5]	-1.8* [-3.5,-0.1]	-0.6 [-2.7,1.5]
index	neighbourhood poverty: high (**wealthy**)	-0.6 [-1.8,0.6]	0.0 [-2.0,2.0]	-1.2 [-2.8,0.3]	-1.0 [-2.9,1.0]
index	vulnerable (**not vulnerable**)	-0.6 [-2.3,1.1]	-1.3 [-3.9,1.2]	0.8 [-1.5,3.1]	-1.1 [-4.3,2.1]
index	living: single house (**building**)	0.1 [-1.3,1.5]	-1.5 [-3.7,0.7]	-0.5 [-2.1,1.1]	-0.6 [-2.7,1.6]
index	living: collective structure (**building**)	1.4 [-1.2,4.0]	-1.7 [-6.3,2.9]	-4.5* [-8.0,-0.9]	0.5 [-4.2,5.2]
Index - Contact	intimate/family (**housing**)	-9.2*** [-10.2,-8.2]	-13.5*** [-15.1,-11.8]	-8.2*** [-9.4,-6.9]	-7.9*** [-10.0,-5.8]
Index - Contact	pro/school/daily (**housing**)	-10.0*** [-11.3,-8.7]	-15.3*** [-17.8,-12.7]	-10.8*** [-12.4,-9.1]	-11.7*** [-14.3,-9.0]
Contact	Immunity: previously infected < 6months (**NVNI**)	-10.6*** [-12.9,-8.3]	-26.4*** [-28.2,-24.7]	-30.1*** [-31.7,-28.5]	-31.0*** [-33.1,-28.9]
contact	Immunity: previously infected > 6months (**NVNI**)	-15.9*** [-17.7,-14.2]	-20.6*** [-23.4,-17.8]	-15.4*** [-17.5,-13.4]	-4.8** [-8.2,-1.3]
contact	Immunity: vaccinated < 6 months (**NVNI**)		-13.2*** [-16.0,-10.5]	-9.6*** [-10.8,-8.5]	7.8*** [5.6,9.9]
contact	Immunity: vaccinated > 6 months (**NVNI**)			1.1 [-1.7,3.9]	13.5*** [11.2,15.8]
contact	Immunity: hybrid (**NVNI**)		-20.3*** [-27.7,-12.9]	-20.8*** [-22.1,-19.6]	-17.4*** [-19.8,-15.0]
contact	women (**men**)	0.3 [-0.4,1.0]	-1.0 [-2.2,0.2]	0.1 [-0.8,1.0]	-0.3 [-1.5,0.9]
contact	age: 0-17 (**18-64**)	-11.3*** [-12.1,-10.5]	-8.3*** [-9.7,-6.9]	0.3 [-0.9,1.6]	-3.9*** [-5.4,-2.3]
contact	65+ (**18-64**)	3.6*** [1.7,5.5]	1.6 [-1.5,4.7]	-0.8 [-2.7,1.1]	-7.8*** [-11.2,-4.4]
contact	Number of tests last 90 days: 0 (**1**)	-17.3*** [-18.6,-16.0]	-11.1*** [-12.8,-9.4]	-13.6*** [-15.1,-12.1]	-17.2*** [-18.9,-15.5]
contact	Number of tests last 90 days: 2+ (**1**)	0.9 [-2.2,3.9]	1.5 [-1.4,4.3]	2.3* [0.1,4.4]	-0.5 [-2.5,1.5]

table S10: Coefficients of the multivariate generalized estimating equation [Confidence Interval]- when considering period of predominance of VoCs above 90%, providing the additional effect on the reference secondary attack rate (first line), for the 4 periods of dominance of the variants EU1, alpha, delta, and omicron. Estimates, p values and confidence intervals are produced by a generalized estimating equations linear regression with robust standard errors predicting a binary outcome indicating if the contact was infected by the index or not, using the index cases as cluster and an exchangeable correlation structure. The estimates are based on 38,148 index-contact relations for the EU variant, 15,662 for the alpha variant, 21,360 for the delta variant and 17,692 for the omicron variant. Estimates are adjusted for the index case gender, age, obesity, presence of symptoms, presence of cough, immunity status, neighbourhood socio-economic condition, vulnerability and type of living; the link between the index case and its contacts, and for the contact persons, their gender, age, number of tests performed the six months before the contact date with the index case, and their immunity status. The reference index case - contact relation of this multivariate analysis is the contact between two men of age below 65 living at the same place, the index being not vaccinated not infected (NVNI), not obese, living in a wealthy neighbourhood and being not a vulnerable person, living in a housing building, and the contact person being a NVNI adult men who performed one SARS-CoV-2 test during the last 6 month preceding the contact. The reference category for each categorical variable is indicated in bold in parenthesis. The left column indicates if the variable concerns the index case, the contact, or their relation. p values are indicated with *. *: $0.01<p<0.05$, **: $0.001<p<0.01$, ***: $p<0.001$

Misclassification of community cases

In order to control for potential misclassification of community cases, we reproduced the multivariable analysis but considering that the contact is positive if it is tested positive between 4 and 10 days after the last encounter with its index case. This sensitivity analysis has for objective to test for a potential effect of index-contact case misclassification: in the main analysis, we suppose that the index case is the one receiving the positive test the first, and the positive contact the one receiving the positive test after. But the reality could be the opposite. By considering contact positive only with a delay of at least 3 days, we lower this risk of misclassification. The results are presented in Figure S11 and table S11. The reference SAR is of course lower than in the main study, because less contacts comply with the new restrictive positivity criteria, but the relative influence of the factor remains similar to the main analysis.

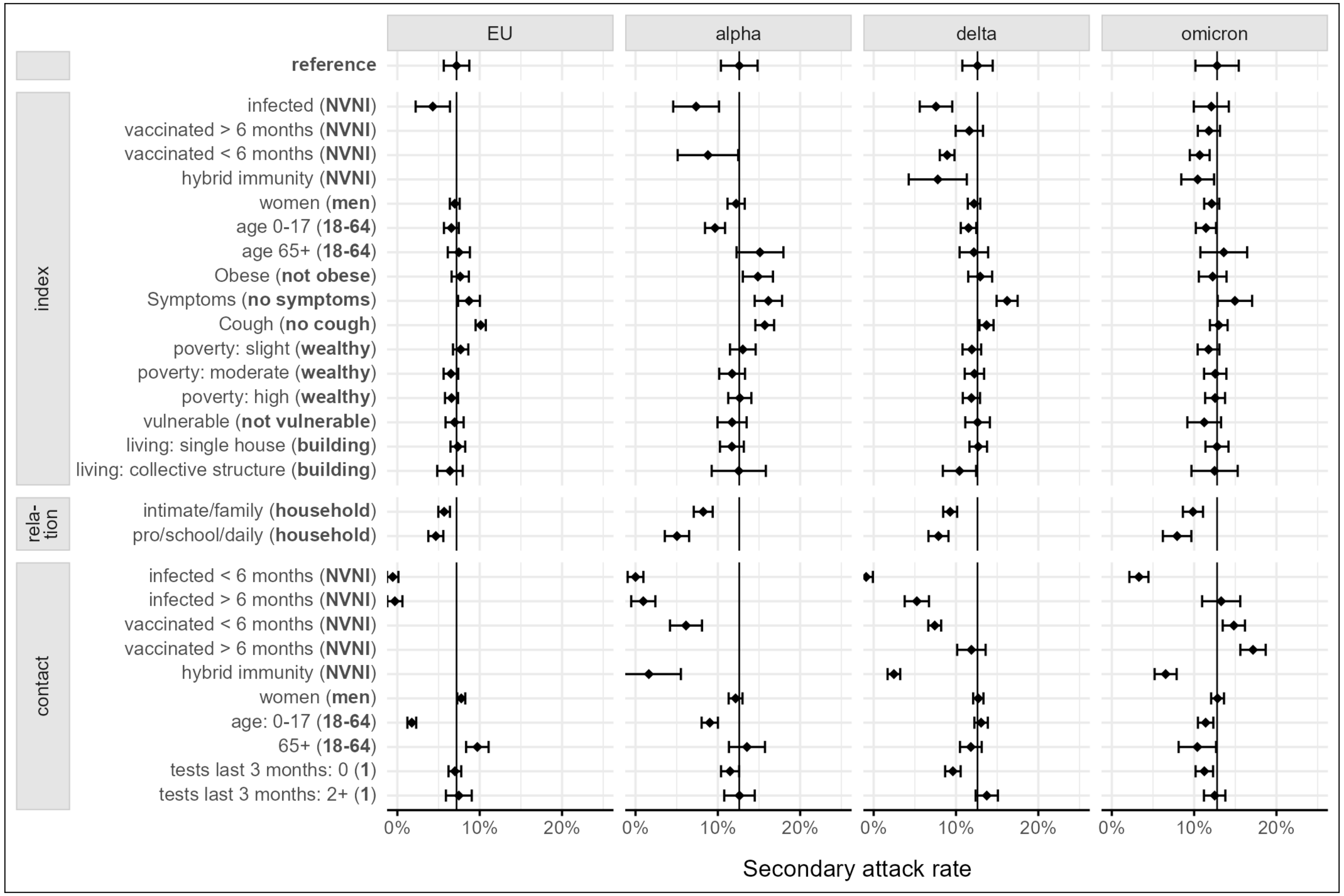

Figure S11: Adjusted Secondary Attack Rate (SAR) stratified per variant when considering that the contact is positive if it is tested positive between 4 and 10 days after the last encounter with its index case, stratified per variant dominance (EU1, alpha, delta and omicron), with the reference value indicated with a vertical line (reference of each covariate is indicated in bold in parenthesis). Estimates and confidence intervals are produced by a generalized estimating equations linear regression with robust standard errors predicting a binary outcome indicating if the contact was infected between 4 and 10 days after the last encounter with its index case, using the index cases as cluster and an exchangeable correlation structure. The estimates are based on 42,295 index-contact relations for the EU variant, 20,311 for the alpha variant, 27,260 for the delta variant and 21,808 for the omicron variant. Estimates are adjusted for the index case gender, age, obesity, presence of symptoms, presence of cough, immunity status, neighbourhood socio-economic condition, vulnerability and type of living; the link between the index case and its contacts, and for the contact persons, their gender, age, number of tests performed the six months before the contact date with the index case, and their immunity status. The reference index case - contact relation of

this multivariate analysis is the contact between two men of age below 65 living at the same place, the index being not vaccinated not infected (NVNI), not obese, living in a wealthy neighbourhood and being not a vulnerable person, living in a housing building, and the contact person being a NVNI adult men who performed one SARS-CoV-2 test during the last 6 month preceding the contact.

		EU1	alpha	delta	omicron
	Reference	7.2*** [5.6,8.7]	12.6*** [10.4,14.8]	12.6*** [10.8,14.5]	12.8*** [10.2,15.4]
index	Immunity: previously infected (**NVNI**)	-2.9** [-5.0,-0.8]	-5.2*** [-8.0,-2.5]	-5.0*** [-7.0,-3.1]	-0.7 [-2.8,1.4]
index	Immunity: vaccinated < 6 months (**NVNI**)			-1.0 [-2.7,0.7]	-1.0 [-2.3,0.4]
index	Immunity: vaccinated > 6 months (**NVNI**)		-3.8* [-7.5,-0.1]	-3.7*** [-4.6,-2.8]	-2.1*** [-3.3,-0.9]
index	Hybrid immunity (**NVNI**)		10.1 [-9.2,29.4]	-4.8** [-8.4,-1.3]	-2.4* [-4.4,-0.4]
index	women (**men**)	-0.2 [-0.8,0.4]	-0.4 [-1.4,0.7]	-0.4 [-1.2,0.3]	-0.7 [-1.6,0.3]
index	age 0-17 (**18-64**)	-0.6 [-1.5,0.3]	-2.9*** [-4.2,-1.7]	-1.1* [-2.0,-0.2]	-1.4* [-2.6,-0.1]
index	age 65+ (**18-64**)	0.3 [-1.1,1.6]	2.5 [-0.3,5.4]	-0.4 [-2.2,1.3]	0.8 [-2.0,3.7]
index	Obese (**not obese**)	0.4 [-0.6,1.5]	2.3* [0.4,4.1]	0.3 [-1.1,1.8]	-0.5 [-2.2,1.1]
index	Symptoms (**no symptoms**)	1.5* [0.2,2.8]	3.5*** [1.9,5.2]	3.6*** [2.3,4.9]	2.2* [0.1,4.3]
index	Cough (**no cough**)	2.9*** [2.3,3.6]	3.1*** [2.0,4.2]	1.1* [0.2,1.9]	0.2 [-0.9,1.3]
index	neighbourhood poverty: slight (**wealthy**)	0.5 [-0.4,1.4]	0.4 [-1.1,2.0]	-0.7 [-1.8,0.4]	-1.0 [-2.4,0.3]
index	neighbourhood poverty: moderate (**wealthy**)	-0.7 [-1.6,0.2]	-0.9 [-2.4,0.7]	-0.4 [-1.6,0.8]	-0.2 [-1.6,1.1]
index	neighbourhood poverty: high (**wealthy**)	-0.6 [-1.4,0.2]	0.1 [-1.3,1.5]	-0.7 [-1.8,0.3]	-0.2 [-1.5,1.0]
index	vulnerable (**not vulnerable**)	-0.2 [-1.3,0.9]	-0.9 [-2.6,0.9]	0.0 [-1.5,1.5]	-1.6 [-3.6,0.5]
index	living: single house (**building**)	0.2 [-0.7,1.1]	-0.9 [-2.3,0.6]	0.1 [-1.0,1.2]	-0.0 [-1.4,1.4]
index	living: collective structure (**building**)	-0.8 [-2.3,0.8]	-0.1 [-3.3,3.2]	-2.2* [-4.2,-0.2]	-0.3 [-3.1,2.5]
Index - Contact	intimate/family (**housing**)	-1.5*** [-2.2,-0.8]	-4.4*** [-5.5,-3.2]	-3.3*** [-4.2,-2.5]	-2.9*** [-4.2,-1.7]
Index - Contact	pro/school/daily (**housing**)	-2.5*** [-3.4,-1.6]	-7.6*** [-9.0,-6.1]	-4.7*** [-6.0,-3.5]	-4.9*** [-6.6,-3.1]
Contact	Immunity: previously infected < 6months (**NVNI**)	-7.8*** [-8.4,-7.1]	-12.6*** [-13.6,-11.6]	-13.5*** [-14.3,-12.7]	-9.5*** [-10.7,-8.3]
contact	Immunity: previously infected > 6months (**NVNI**)	-7.5*** [-8.4,-6.6]	-11.7*** [-13.1,-10.2]	-7.4*** [-8.8,-5.9]	0.5 [-1.8,2.8]
contact	Immunity: vaccinated < 6 months (**NVNI**)		-6.5*** [-8.4,-4.5]	-5.2*** [-6.0,-4.4]	2.0** [0.7,3.4]
contact	Immunity: vaccinated > 6 months (**NVNI**)			-0.7 [-2.5,1.0]	4.4*** [2.8,5.9]
contact	Immunity: hybrid (**NVNI**)		-11.0*** [-14.9,-7.1]	-10.2*** [-10.9,-9.4]	-6.3*** [-7.6,-4.9]
contact	women (**men**)	0.6* [0.1,1.1]	-0.4 [-1.3,0.4]	0.1 [-0.5,0.7]	0.1 [-0.7,0.8]
contact	age: 0-17 (**18-64**)	-5.4*** [-6.0,-4.9]	-3.6*** [-4.6,-2.6]	0.4 [-0.4,1.3]	-1.4** [-2.3,-0.5]
contact	65+ (**18-64**)	2.5*** [1.2,3.9]	0.9 [-1.3,3.1]	-0.8 [-2.1,0.5]	-2.4* [-4.7,-0.1]
contact	Number of tests last 90 days: 0 (**1**)	-0.2 [-1.0,0.6]	-1.1* [-2.2,-0.0]	-3.0*** [-3.9,-2.1]	-1.6** [-2.6,-0.5]
contact	Number of tests last 90 days: 2+ (**1**)	0.3 [-1.3,1.8]	0.0 [-1.8,1.9]	1.1 [-0.2,2.5]	-0.3 [-1.6,1.0]

table S11: Coefficients of the multivariate generalized estimating equation [Confidence Interval] when considering that the contact is positive if it is tested positive between 4 and 10 days after the last encounter with its index case, providing the additional effect on the reference secondary attack rate (first line), for the 4 periods of dominance of the variants EU1, alpha, delta, and omicron. Estimates, p values and confidence intervals are produced by a generalized estimating equations linear regression with robust standard errors predicting a binary outcome indicating if the contact was infected between 4 and 10 days after the last encounter with its index case, using the index cases as cluster and an exchangeable correlation structure. The estimates are based on 42,295 index-contact relations for the EU variant, 20,311 for the alpha variant, 27,260 for the delta variant and 21,808 for the omicron variant. Estimates are adjusted for the index case gender, age, obesity, presence of symptoms, presence of cough, immunity status, neighbourhood socio-economic condition, vulnerability and type of living; the link between the index case and its contacts, and for the contact persons, their gender, age, number of tests performed the six months before the contact date with the index case, and their immunity status. The reference index case - contact relation of this multivariate analysis is the contact between two men of age below 65 living at the same place, the index being not vaccinated not infected (NVNI), not obese, living in a wealthy neighbourhood and being not a vulnerable person, living in a housing building, and the contact person being a NVNI adult men who performed one SARS-CoV-2 test during the last 6 month preceding the contact. The reference category for each categorical variable is indicated in bold in parenthesis. The left column indicates if the variable concerns the index case, the contact, or their relation. p values are indicated with *. *: 0.01<p<0.05, **:0.001<p<0.01, ***: p<0.001

Misclassification of primary case

In order to control for potential misclassification of primary case, we reproduced the multivariable analysis but restricting the data to household with only one contact. This sensitivity analysis has for objective to test for a potential effect misclassification of primary case: in the main analysis, we suppose that the index case is the one receiving the positive test the first, and the positive contact the one receiving the positive test after, when the contact could actually be contaminated by a third party, contact of the index case. By restricting the analysis to households with only one contact, we avoid this risk of misclassification, and obtain 8669 index-contact relations. The results are presented in Figure S12 and table S12.

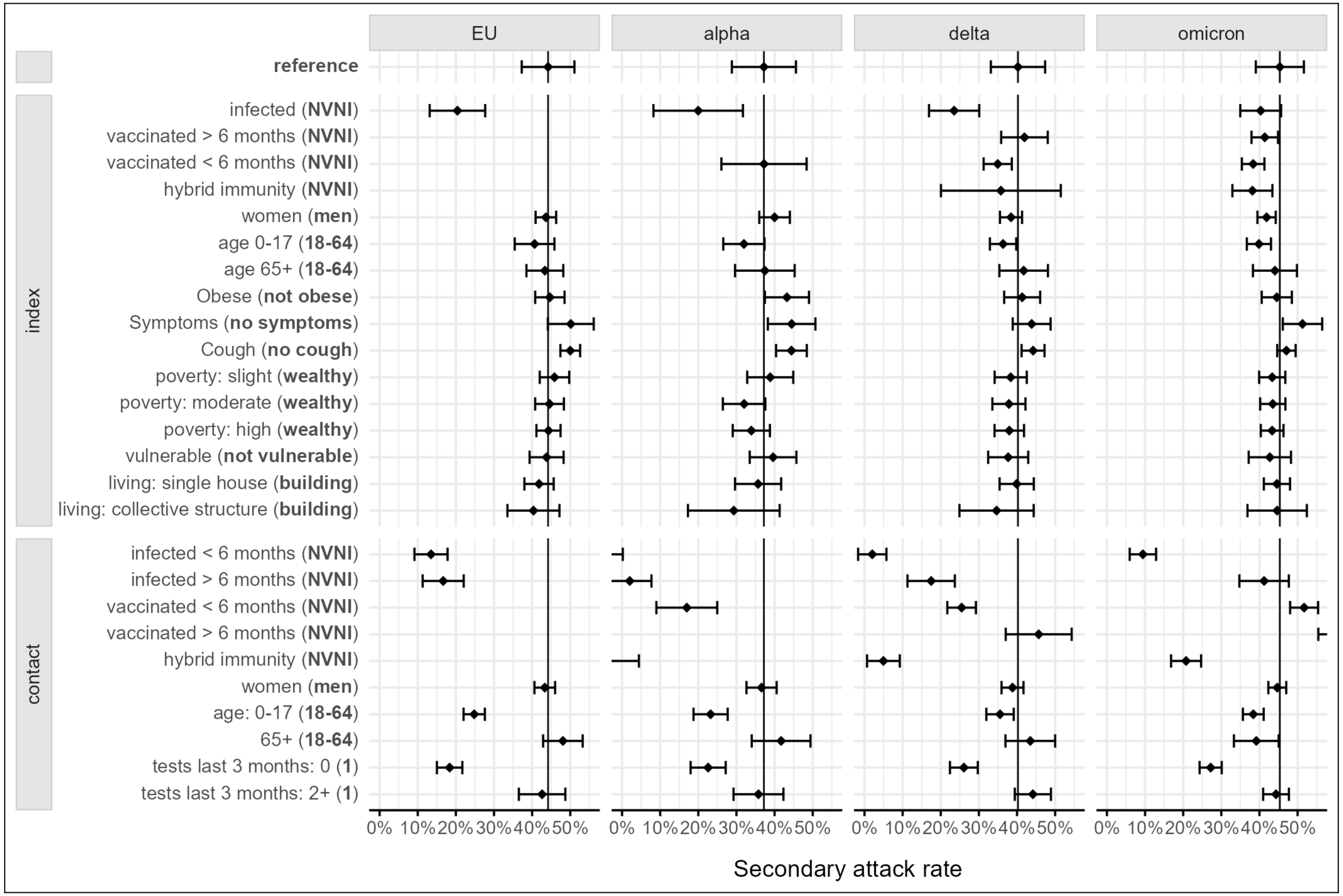

Figure S12: Estimated Adjusted Secondary Attack Rate stratified per variant when restricting the data to household with only one contact, stratified per variant dominance (EU1, alpha, delta and omicron), with the reference value indicated with a vertical line (reference of each covariate is indicated in bold in parenthesis). Estimates and confidence intervals are produced by a generalized estimating equations linear regression with robust standard errors predicting a binary outcome indicating if the contact was infected before 10 days after the last encounter with its index case, using the index cases as cluster and an exchangeable correlation structure. The estimates are based on 2,783 index-contact relations for the EU variant, 2,016 for the alpha variant, 2,266 for the delta variant and 1,604 for the omicron variant. Estimates are adjusted for the index case gender, age, obesity, presence of symptoms, presence of cough, immunity status, neighbourhood socio-economic condition, vulnerability and type of living; the link between the index case and its contacts, and for the contact persons, their gender, age, number of tests performed the six months before the contact date with the index case, and their immunity status. The reference index case - contact relation of this multivariate analysis is the contact between two men of age below 65 living at the same place, the index being not vaccinated not infected (NVNI), not obese, living in a wealthy neighbourhood and being not a vulnerable person, living in a housing building, and the contact person being a NVNI adult men who performed one SARS-CoV-2 test during the last 6 month preceding the contact.

		EU1	alpha	delta	omicron
	Reference	44.2*** [37.3,51.1]	37.2*** [28.8,45.6]	40.3*** [33.1,47.4]	45.3*** [39.0,51.7]
index	Immunity: previously infected (**NVNI**)	-23.8*** [-31.1,-16.5]	-17.2** [-29.0,-5.5]	-16.8*** [-23.3,-10.2]	-5.0 [-10.4,0.3]
index	Immunity: vaccinated < 6 months (**NVNI**)			1.7 [-4.4,7.8]	-4.0* [-7.4,-0.5]
index	Immunity: vaccinated > 6 months (**NVNI**)		0.0 [-11.2,11.2]	-5.3** [-9.0,-1.6]	-7.0*** [-10.0,-4.0]
index	Hybrid immunity (**NVNI**)		35.7 [-15.9,87.2]	-4.5 [-20.2,11.2]	-7.2** [-12.4,-2.0]
index	women (**men**)	-0.6 [-3.3,2.1]	2.8 [-1.2,6.8]	-1.8 [-4.7,1.1]	-3.5** [-5.9,-1.1]
index	age 0-17 (**18-64**)	-3.5 [-8.8,1.7]	-5.2 [-10.7,0.2]	-3.9* [-7.4,-0.5]	-5.5*** [-8.7,-2.3]
index	age 65+ (**18-64**)	-0.8 [-5.7,4.0]	0.2 [-7.6,8.1]	1.5 [-4.9,7.9]	-1.3 [-7.1,4.5]
index	Obese (**not obese**)	0.5 [-3.4,4.3]	6.0* [0.3,11.8]	1.1 [-3.6,5.8]	-0.8 [-4.8,3.1]
index	Symptoms (**no symptoms**)	6.0 [-0.1,12.0]	7.3* [1.0,13.5]	3.6 [-1.4,8.6]	5.9* [0.8,11.1]
index	Cough (**no cough**)	5.8*** [3.2,8.4]	7.2*** [3.2,11.2]	4.0* [0.9,7.0]	1.7 [-0.7,4.1]
index	neighbourhood poverty: slight (**wealthy**)	1.7 [-2.2,5.5]	1.7 [-4.4,7.7]	-1.9 [-6.1,2.3]	-2.0 [-5.4,1.4]
index	neighbourhood poverty: moderate (**wealthy**)	0.4 [-3.4,4.1]	-5.2 [-10.8,0.4]	-2.4 [-6.7,2.0]	-1.9 [-5.2,1.5]
index	neighbourhood poverty: high (**wealthy**)	0.1 [-3.1,3.3]	-3.3 [-8.2,1.6]	-2.3 [-6.1,1.6]	-2.0 [-5.0,1.0]
index	vulnerable (**not vulnerable**)	-0.4 [-4.9,4.1]	2.4 [-3.7,8.5]	-2.6 [-7.8,2.7]	-2.6 [-8.2,2.9]
index	living: single house (**building**)	-2.4 [-6.2,1.5]	-1.5 [-7.6,4.5]	-0.3 [-4.8,4.1]	-0.8 [-4.2,2.7]
index	living: collective structure (**building**)	-3.8 [-10.7,3.0]	-7.9 [-19.9,4.1]	-5.6 [-15.4,4.1]	-0.7 [-8.5,7.1]
Contact	Immunity: previously infected < 6months (**NVNI**)	-30.7*** [-35.0,-26.4]	-41.2*** [-45.3,-37.0]	-38.2*** [-41.9,-34.5]	-35.9*** [-39.4,-32.5]
contact	Immunity: previously infected > 6months (**NVNI**)	-27.5*** [-32.9,-22.1]	-35.2*** [-40.9,-29.5]	-22.8*** [-29.0,-16.6]	-4.1 [-10.6,2.4]
contact	Immunity: vaccinated < 6 months (**NVNI**)		-20.2*** [-28.2,-12.2]	-14.8*** [-18.5,-11.0]	6.4*** [2.7,10.0]
contact	Immunity: vaccinated > 6 months (**NVNI**)			5.4 [-3.2,14.1]	14.2*** [10.1,18.3]
contact	Immunity: hybrid (**NVNI**)		-45.8*** [-58.9,-32.8]	-35.3*** [-39.6,-31.0]	-24.6*** [-28.5,-20.7]
contact	women (**men**)	-0.9 [-3.6,1.8]	-0.6 [-4.6,3.3]	-1.4 [-4.3,1.4]	-0.7 [-3.0,1.7]
contact	age: 0-17 (**18-64**)	-19.4*** [-22.2,-16.6]	-14.0*** [-18.5,-9.5]	-4.7* [-8.3,-1.1]	-7.0*** [-9.7,-4.2]
contact	65+ (**18-64**)	3.9 [-1.3,9.1]	4.5 [-3.2,12.2]	3.2 [-3.3,9.7]	-6.2* [-12.0,-0.3]
contact	Number of tests last 90 days: 0 (**1**)	-25.8*** [-29.2,-22.5]	-14.6*** [-19.2,-10.0]	-14.2*** [-17.8,-10.5]	-18.1*** [-21.0,-15.2]
contact	Number of tests last 90 days: 2+ (**1**)	-1.6 [-7.7,4.5]	-1.4 [-8.0,5.1]	3.9 [-0.8,8.6]	-1.0 [-4.4,2.4]

table S12: Coefficients of the multivariate generalized estimating equation [Confidence Interval] when restricting the data to household with only one contact, providing the additional effect on the reference secondary attack rate (first line), for the 4 periods of dominance of the variants EU1, alpha, delta, and omicron. Estimates, p values and confidence intervals are produced by a generalized estimating equations linear regression with robust standard errors predicting a binary outcome indicating if the contact was infected before 10 days after the last encounter with its index case, using the index cases as cluster and an exchangeable correlation structure. The estimates are based on 2,783 index-contact relations for the EU variant, 2,016 for the alpha variant, 2,266 for the delta variant and 1,604 for the omicron variant. Estimates are adjusted for the index case gender, age, obesity, presence of symptoms, presence of cough, immunity status, neighbourhood socio-economic condition, vulnerability and type of living; the link between the index case and its contacts, and for the contact persons, their gender, age, number of tests performed the six months before the contact date with the index case, and their immunity status. The reference index case - contact relation of this multivariate analysis is the contact between two men of age below 65 living at the same place, the index being not vaccinated not infected (NVNI), not obese, living in a wealthy neighbourhood and being not a vulnerable person, living in a housing building, and the contact person being a NVNI adult men who performed one SARS-CoV-2 test during the last 6 month preceding the contact. The reference category for each categorical variable is indicated in bold in parenthesis. The left column indicates if the variable concerns the index case, the contact, or their relation. p values are indicated with *. *: $0.01<p<0.05$, **:$0.001<p<0.01$, ***: $p<0.001$

Misclassification of tertiary case

In order to control for potential misclassification of tertiary case, we reproduced the multivariable analysis but considering only contacts that were set in quarantine at maximum de day after their last contact with the index case. This sensitivity analysis has for objective to test for a potential misclassification of community case: in the main analysis, we suppose that the contacts are infected by the index cases, whereas they could by infected by other persons. By considering contact set in quarantine right after their last contact with the index, we lower this risk. The obtained dataset is composed of 38,277 index-contact relations. The results are presented in Figure S13 and table S13.

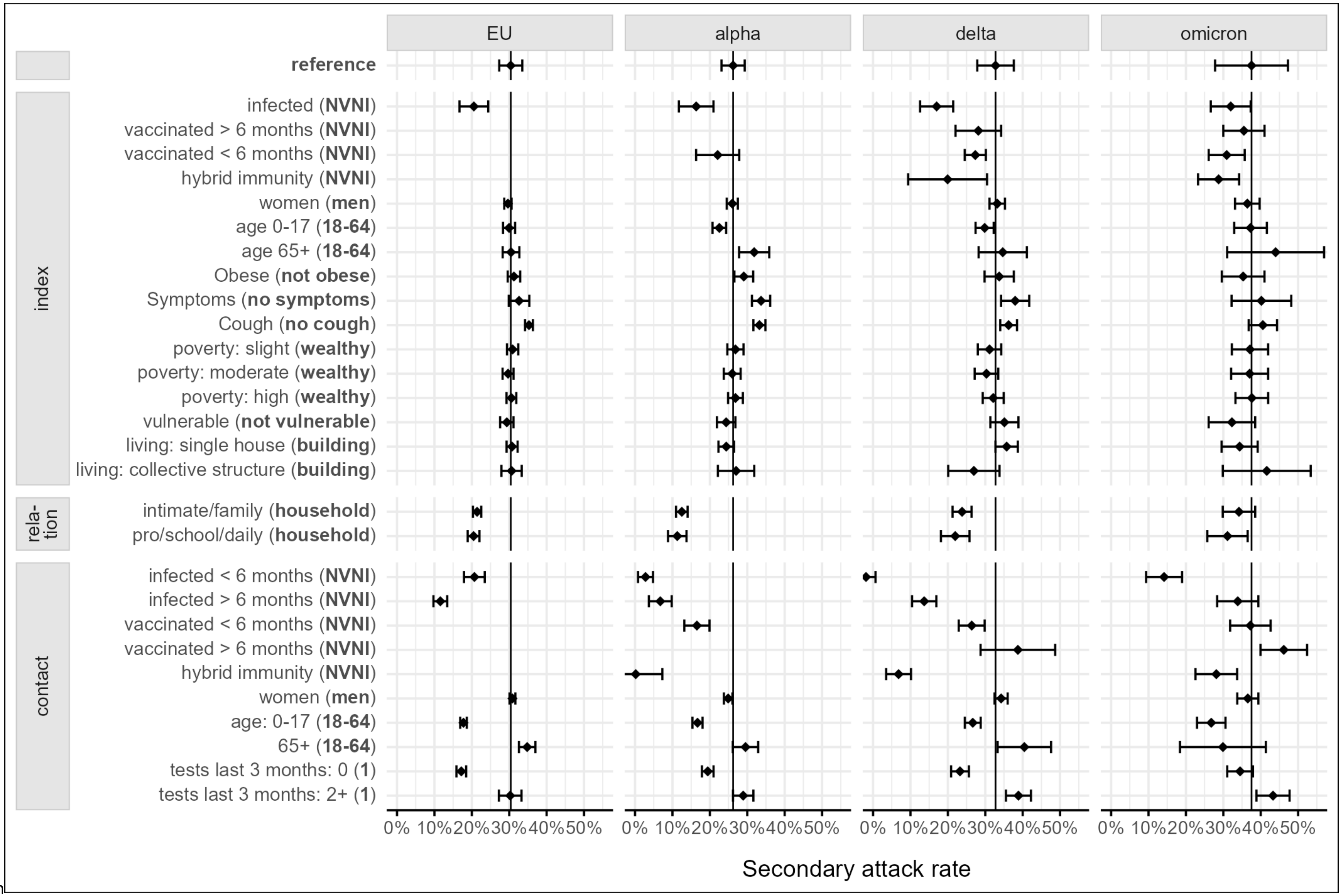

Figure S13: Adjusted Secondary Attack Rate (SAR) stratified per variant when restricting the data to contacts set in quarantine at maximum one day after their last encounter with the index case, stratified per variant dominance (EU1, alpha, delta and omicron), with the reference value indicated with a vertical line (reference of each covariate is indicated in bold in parenthesis). Estimates and confidence intervals are produced by a generalized estimating equations linear regression with robust standard errors predicting a binary outcome indicating if the contact was infected before 10 days after the last encounter with its index case, using the index cases as cluster and an exchangeable correlation structure. The estimates are based on 31,429 index-contact relations for the EU variant, 16,699 for the alpha variant, 7,838 for the delta variant and 3,260 for the omicron variant. Estimates are adjusted for the index case gender, age, obesity, presence of symptoms, presence of cough, immunity status, neighbourhood socio-economic condition, vulnerability and type of living; the link between the index case and its contacts, and for the contact persons, their gender, age, number of tests performed the six months before the contact date with the index case, and their immunity status. The reference index case - contact relation of this multivariate analysis is the contact between two men of age below 65 living at the same place, the index being not vaccinated not infected (NVNI), not obese, living in a wealthy neighbourhood and being not a vulnerable person, living in a housing building, and the contact person being a NVNI adult men who performed one SARS-CoV-2 test during the last 6 month preceding the contact.

		EU1	alpha	delta	omicron
	Reference	30.4*** [27.3,33.5]	26.2*** [23.1,29.3]	32.7*** [27.9,37.6]	37.5*** [27.8,47.3]
index	Immunity: previously infected (**NVNI**)	-9.9*** [-13.7,-6.0]	-9.9*** [-14.5,-5.2]	-15.7*** [-20.2,-11.3]	-5.6* [-10.9,-0.2]
index	Immunity: vaccinated < 6 months (**NVNI**)			-4.6 [-10.7,1.5]	-2.0 [-7.6,3.5]
index	Immunity: vaccinated > 6 months (**NVNI**)		-4.1 [-9.9,1.6]	-5.4*** [-8.2,-2.6]	-6.6** [-11.4,-1.8]
index	Hybrid immunity (**NVNI**)		23.6 [-15.1,62.3]	-12.8* [-23.4,-2.3]	-8.8** [-14.3,-3.3]
index	women (**men**)	-0.8 [-1.7,0.2]	-0.2 [-1.7,1.3]	0.5 [-1.6,2.5]	-1.1 [-4.4,2.2]
index	age 0-17 (**18-64**)	-0.5 [-2.1,1.2]	-3.7*** [-5.5,-1.9]	-2.9* [-5.3,-0.5]	-0.3 [-4.6,4.1]
index	age 65+ (**18-64**)	0.1 [-2.2,2.3]	5.6** [1.6,9.7]	1.9 [-4.5,8.4]	6.4 [-6.5,19.4]
index	Obese (**not obese**)	0.9 [-0.8,2.5]	2.9* [0.3,5.4]	1.0 [-2.9,4.9]	-2.2 [-7.9,3.5]
index	Symptoms (**no symptoms**)	2.2 [-0.5,5.0]	7.5*** [5.0,9.9]	5.2** [1.5,9.0]	2.6 [-5.3,10.6]
index	Cough (**no cough**)	4.9*** [3.8,5.9]	7.0*** [5.4,8.6]	3.5** [1.3,5.7]	3.0 [-0.8,6.8]
index	neighbourhood poverty: slight (**wealthy**)	0.5 [-1.0,2.0]	0.6 [-1.5,2.8]	-1.6 [-4.7,1.5]	-0.4 [-5.3,4.4]
index	neighbourhood poverty: moderate (**wealthy**)	-0.7 [-2.2,0.7]	-0.2 [-2.5,2.0]	-2.4 [-5.6,0.7]	-0.5 [-5.5,4.4]
index	neighbourhood poverty: high (**wealthy**)	0.2 [-1.1,1.5]	0.6 [-1.3,2.6]	-0.6 [-3.4,2.2]	0.1 [-4.3,4.5]
index	vulnerable (**not vulnerable**)	-1.1 [-2.8,0.7]	-1.9 [-4.3,0.6]	2.4 [-1.4,6.1]	-5.2 [-11.5,1.0]
index	living: single house (**building**)	0.4 [-1.1,1.8]	-1.8 [-3.9,0.3]	3.0 [-0.0,6.0]	-3.2 [-8.0,1.6]
index	living: collective structure (**building**)	0.2 [-2.5,2.9]	0.8 [-4.1,5.6]	-5.8 [-12.7,1.1]	4.1 [-7.7,15.8]
Index - Contact	intimate/family (**housing**)	-9.0*** [-10.1,-7.9]	-13.7*** [-15.3,-12.2]	-9.0*** [-11.5,-6.4]	-3.4 [-7.7,1.0]
Index - Contact	pro/school/daily (**housing**)	-9.9*** [-11.5,-8.4]	-14.9*** [-17.4,-12.5]	-10.8*** [-14.6,-7.0]	-6.4* [-11.8,-1.0]
Contact	Immunity: previously infected < 6months (**NVNI**)	-9.7*** [-12.5,-6.9]	-23.4*** [-25.4,-21.4]	-34.6*** [-37.2,-32.1]	-23.4*** [-28.2,-18.6]
contact	Immunity: previously infected > 6months (**NVNI**)	-18.8*** [-20.7,-17.0]	-19.5*** [-22.5,-16.4]	-19.1*** [-22.3,-15.8]	-3.7 [-9.2,1.8]
contact	Immunity: vaccinated < 6 months (**NVNI**)		-9.7*** [-13.1,-6.3]	-6.4*** [-9.8,-2.9]	-0.3 [-5.7,5.1]
contact	Immunity: vaccinated > 6 months (**NVNI**)			6.0 [-4.0,16.0]	8.6** [2.4,14.9]
contact	Immunity: hybrid (**NVNI**)		-26.0*** [-33.1,-18.9]	-25.9*** [-29.2,-22.6]	-9.4*** [-15.0,-3.8]
contact	women (**men**)	0.5 [-0.3,1.2]	-1.3* [-2.5,-0.2]	1.5 [-0.3,3.2]	-1.0 [-3.8,1.8]
contact	age: 0-17 (**18-64**)	-12.6*** [-13.5,-11.8]	-9.5*** [-10.9,-8.2]	-6.1*** [-8.2,-4.0]	-10.8*** [-14.6,-7.0]
contact	65+ (**18-64**)	4.4*** [2.2,6.6]	3.3 [-0.1,6.7]	7.7* [0.6,14.8]	-7.6 [-19.1,3.8]
contact	Number of tests last 90 days: 0 (**1**)	-13.2*** [-14.5,-11.9]	-6.8*** [-8.3,-5.2]	-9.5*** [-11.9,-7.1]	-3.1 [-6.5,0.4]
contact	Number of tests last 90 days: 2+ (**1**)	-0.1 [-3.2,2.9]	2.7 [-0.0,5.4]	6.1*** [2.8,9.5]	5.7* [1.3,10.2]

table S13: Estimated coefficients of the multivariate generalized estimating equation [Confidence Interval] when restricting the data to contacts set in quarantine at maximum one day after their last encounter with the index case, providing the additional effect on the reference secondary attack rate (first line), for the 4 periods of dominance of the variants EU1, alpha, delta, and omicron. Estimates and confidence intervals are produced by a generalized estimating equations linear regression with robust standard errors predicting a binary outcome indicating if the contact was infected before 10 days after the last encounter with its index case, using the index cases as cluster and an exchangeable correlation structure. The estimates are based on 31,429 index-contact relations for the EU variant, 16,699 for the alpha variant, 7,838 for the delta variant and 3,260 for the omicron variant. Estimates are adjusted for the index case gender, age, obesity, presence of symptoms, presence of cough, immunity status, neighbourhood socio-economic condition, vulnerability and type of living; the link between the index case and its contacts, and for the contact persons, their gender, age, number of tests performed the six months before the contact date with the index case, and their immunity status. The reference index case - contact relation of this multivariate analysis is the contact between two men of age below 65 living at the same place, the index being not vaccinated not infected (NVNI), not obese, living in a wealthy neighbourhood and being not a vulnerable person, living in a housing building, and the contact person being a NVNI adult men who performed one SARS-CoV-2 test during the last 6 month preceding the contact. The reference category for each categorical variable is indicated in bold in parenthesis. The left column indicates if the variable concerns the index case, the contact, or their relation. p values are indicated with *. *: $0.01<p<0.05$, **: $0.001<p<0.01$, ***: $p<0.001$

Methods

Background information

Evolution in Time of cases, tests and COVID19 deaths

Supplementary figure S14 provides the evolution in time of the daily number of COVID19 cases, number of SarS-CoV-2 tests and of COVID19 deaths (confirmed by one physician and certified by the state authority) for the studied period.

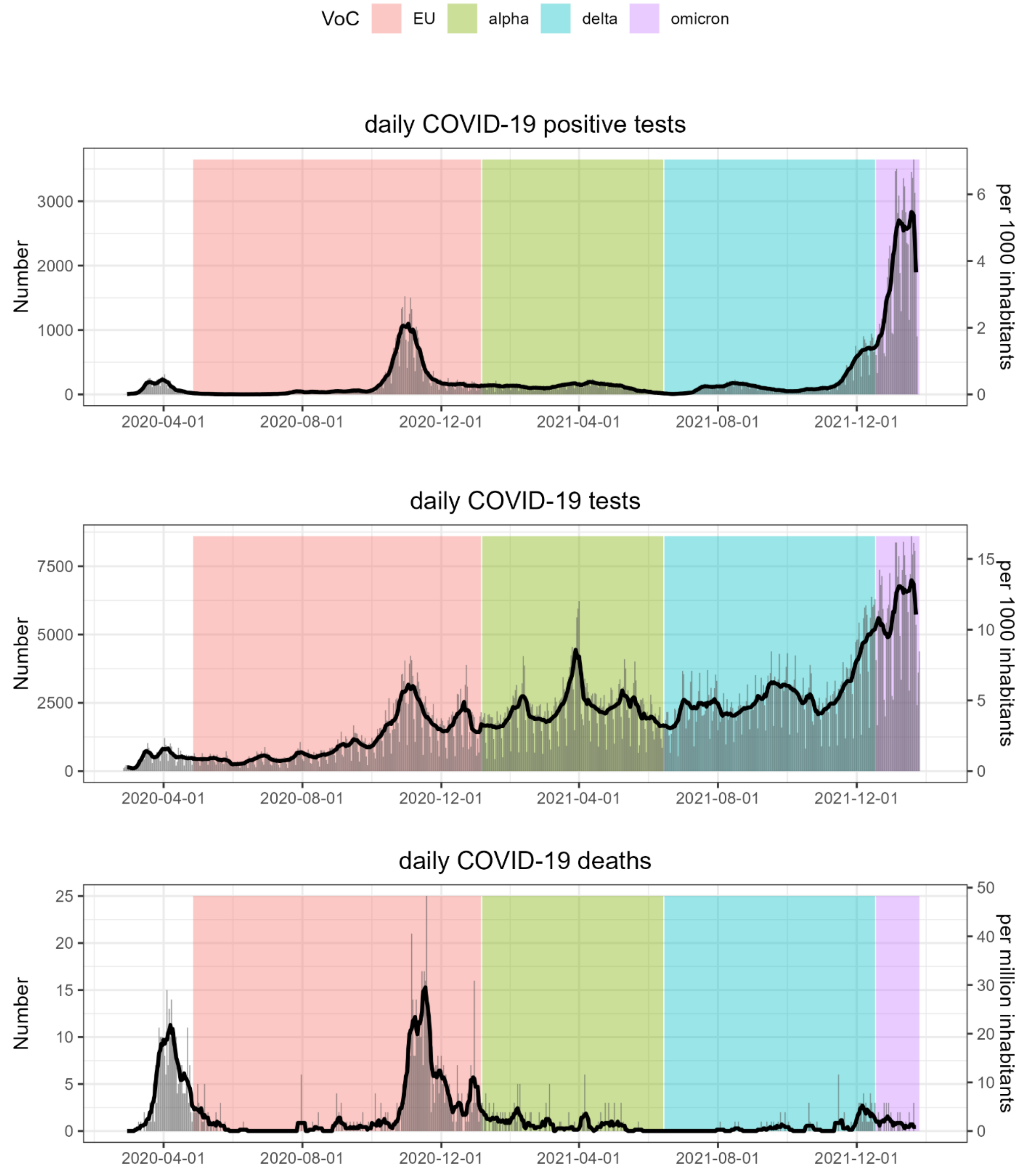

Supplementary figure S14: Evolution in time of the number of positive SarS-CoV-2 tests (upper panel), of the total number of SarS-CoV-2 tests (middle panel) and number of COVID-19 deaths (lower panel) in Geneva

Non-pharmaceutical interventions

From the first COVID-19 positive case in Geneva (26-02-2020), several non-pharmaceutical interventions (NPI) have been put in place. The list and Figure S15 below summarize the main NPIs from March 2020 to February 2022:

- From 12-03-2020 to 26-06-2021: obligation to work from home
- From 16-03-2020 to 11-05-2020: confinement, including schools closure
- From 16-03-2020 to 30-05-2020: restriction of gathering to 5 people
- From 06-07-2020 to 26-06-2021: obligation to wear a mask in public
- From 21-10-2020 to 22-03-2021: restriction of gathering to 5 people
- From 02-11-2020 to 01-03-2021: closure of non-essential shops
- From 13-09-2021 to 17-02-2022: COVID certificate is mandatory in public places
- From 29-11-2021 to 17-02-2022: obligation to wear a mask in public places

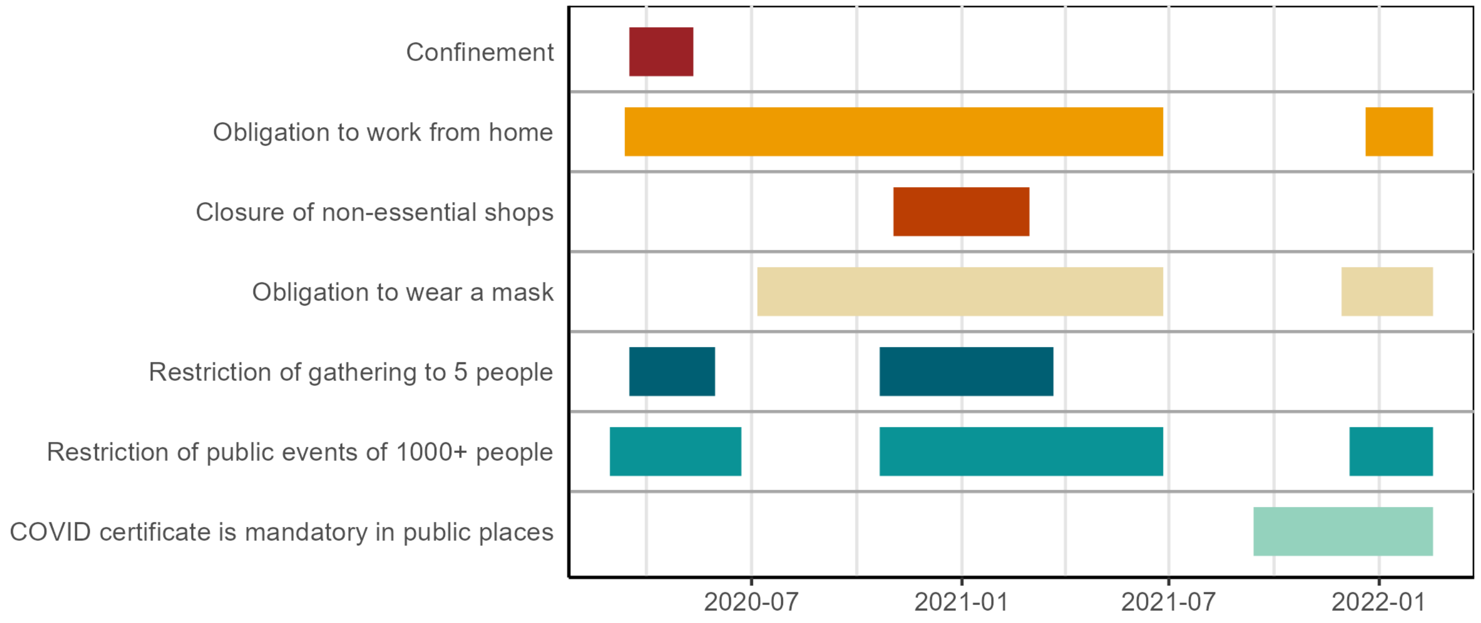

Fig. S15: main non-pharmaceutical interventions (NPI) in Geneva over time. The coloured lines indicate the period during which the NPIs described on the left were active.

During the complete period, both PCR and antigenic tests were free of charge for the population and there were no test accessibility restrictions except for a short period during the first EU1 epidemic wave. Antigenic tests were introduced the 09-11-2020. Note that the testing capacity was saturated during the peak period of the second EU1 wave (end of October 2020) and the Omicron wave (beginning of January 2022).

Vaccination

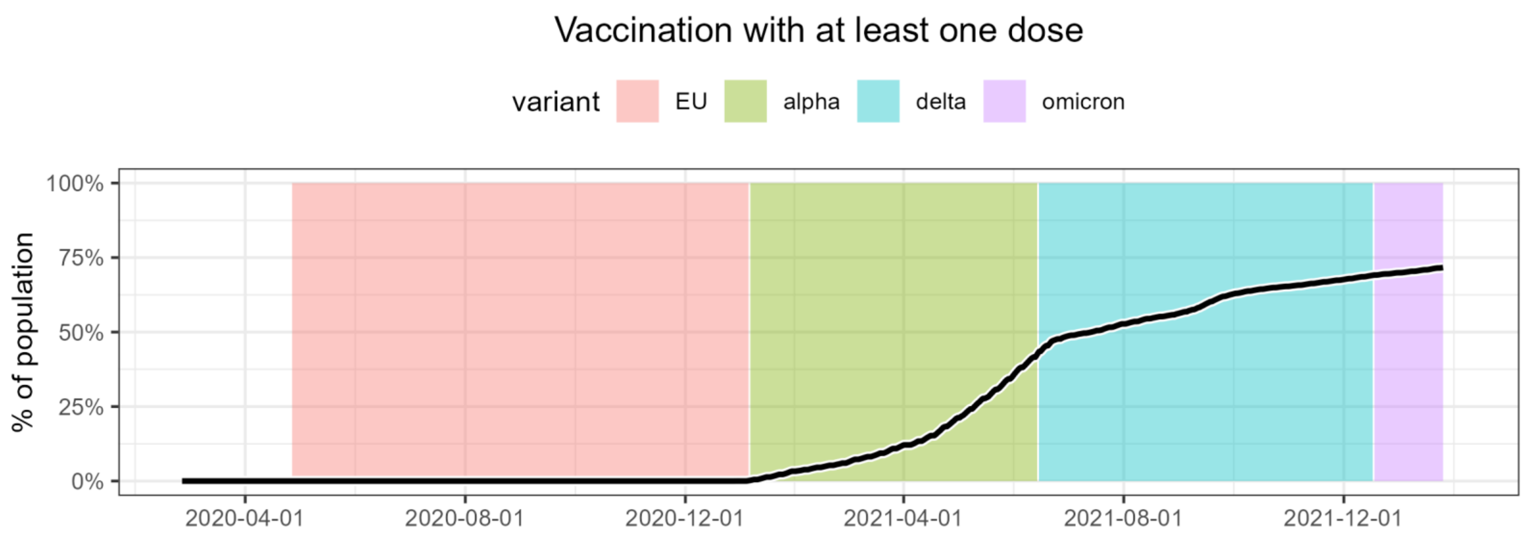

Fig. S16: Evolution in time of the proportion of the population with at least one dose in Geneva

.

In Geneva, the first vaccination centre opened the 25-01-2021 for person aged 75 or more at first. In February, vaccination was extended to vulnerable persons under 75 (immune-compromised persons, diabetic, …) and healthcare collaborators. The targeted population was progressively extended to people over 45 years old (from 12-04-2021), people over 16 years old (from 14-05-2021) and finally to children (from 02-07-2021 and from 12-01-2022 for younger people between 5 to 11 years old respectively). The most administrated vaccine type in Geneva was the RNA-based vaccine type, such as Moderna mRNA-1273 (59.83% of the total administrated vaccine doses in Geneva) and Pfizer BNT162b2 (39.86%), other type of vaccine stands for a minor part: Janssen (0.25%) and Nuvaxovid (0.06%). The final uptake at end of February 2022 was of 71.6% of the population who received at least one dose of vaccine (time evolution in Figure S16).

Link between the index case and its contact

The type of link between the index case and its contact was coded as indicated in the column “initial value” in the original form, and recoded as presented in the “recoded value”:

**Initial value**	**Recoded value**
Live under the same roof	Same roof
Intimate contact	Intimate or familial
Familial relation	Intimate or familial
Professional relation	Other
School	Other
University	Other
Leisure/hobbies	Other
Healthcare	Other
Other	Other

Symptoms

The following symptoms were recording in ARGOS. These symptoms could be filled during any follow-up call. Index case was considered to have a symptom if any of the following was filled during any follow-up. He was considered coughing if he indicated any type of cough during any follow-up call:

- Dry cough
- Productive cough
- Dyspnoea
- Fever
- Chills
- Tiredness or Fatigue
- Muscle and/or joint pain
- Headache
- Runny/stuffy nose
- Sore throat
- Loss of taste/smell
- Conjonctivitis
- Abdominal pain
- Nausea/vomiting
- Diarrhoea
- Skin eruption
- Other
- Digestive symptoms
- Cough
- ear, nose, throat symptoms

"Ear, nose, throat symptoms" is a general variable that was applied at the beginning of the pandemic, when there was no precise idea of the COVID-19 symptoms. Thereafter, runny/stuffy nose, sore throat, and loss of taste/smell symptoms were better characterized, and these categories were added in the list of symptoms. From May 2020, the category "ear, nose, throat symptoms" were no longer used and was replaced by the above-mentioned detailed variables

Personal vulnerability

Personal vulnerability was calculated as a binary variable. A person was considered vulnerable if any of the following applied:

- The address of the person corresponded to a highly subsidised living (habitation bon marché in Switzerland)
- The address of the person corresponded to one of the immigrant hostels in Geneva or one of the institutions to accommodate person without living
- The address of the person was the one of the social administrative tribunals
- The person indicated that he/she did not want to be controlled by the police, because of its illegal situation
- The person declared to have difficulties to make the end meet

Missing data

Missing data were handled using multiple imputation with chained equations (20 samples, 5 iterations) at the person, infection or contact level:

- Missing height, weight, birth year, gender or living socio-economic index were imputed at the person level using predictive mean matching for height, weight and birth year, logistic regression imputation for gender and polytomous regression imputation for the socio-economic index
- Presence or absence of symptoms, and presence or absence of cough was imputed at the infection level using logistic imputation considering also the age, the gender, the body mass index and the SARS-COV2 variant
- Missing contact type and missing immunity/vaccinal status were imputed at the contact level using polytomous regression imputation and considering in the equation the fact of living at the same address, the fact of being contaminated by the index case, and the SARS-cov2 variant.

Population of Geneva

The following table provides for the entire population of Geneva in 2021 the number and percentage of the age categories considered in the study:

age	Total Number	Percentage
0-17	94,085	18.53
18-64	329,611	64.93
65+	83,960	16.54

SARS-CoV-2 variants

The data used to determine the period of Variant predominance are provided by GISEAD and covariant.org, and concern the regions 1 of Switzerland, encompassing Geneva, Valais and Vaud cantons (https://covariants.org/per-country?region=Switzerland). The data were available at the following github repository https://github.com/hodcroftlab/covariants/blob/master/cluster_tables/SwissClusters_data.json and are provided in the gitlab repository of this article. After selecting the data for the region1 of Switzerland and the four variants of interest in our study, we modelled the VoC evolution as a rising and decreasing sigmoid in time. This approach yields the estimations presented in the graph below (estimations are solid lines, points are the experimental data)

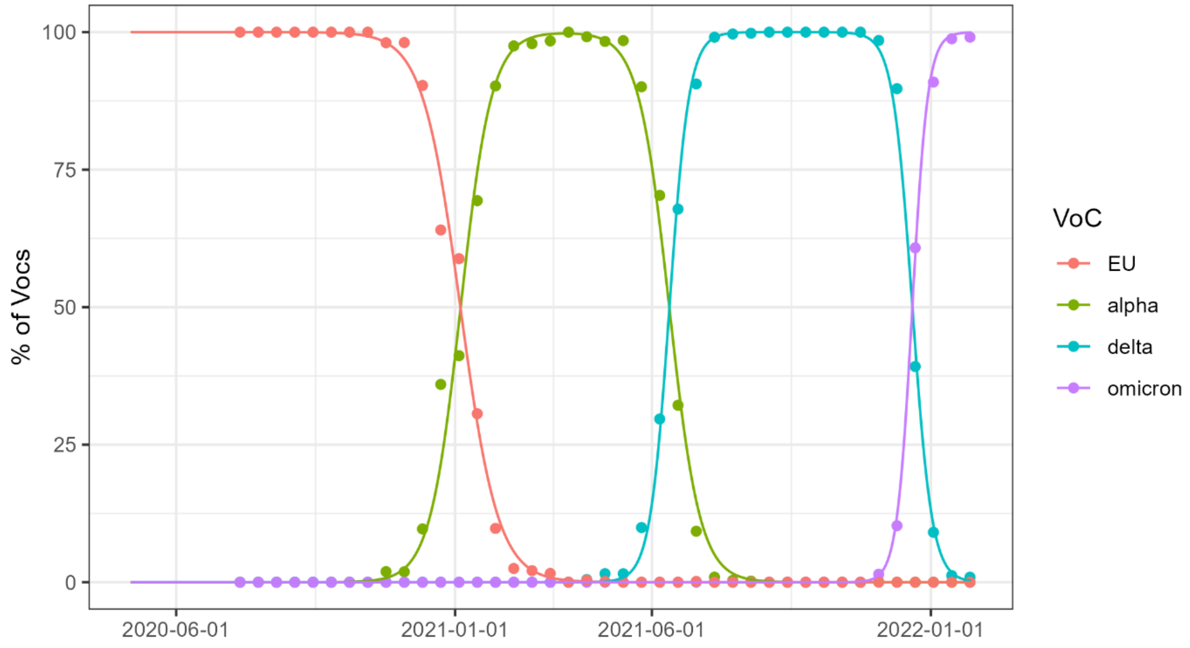

Supplementary figure S17: share of SarS-CoV-2 Variant of Concern (VoC) in the Geneva region: measures from GISEAD (points) and the estimation based on sigmoid functions (solid lines)

From this estimated curve, we determine the dates for two set of dominance threshold:
For 50% dominance:

VoC	EU	alpha	delta	Omicron
Start	01-06-2020	06-01-2021	15-06-2021	18-12-2021
end	05-01-2021	14-06-2021	17-12-2021	01-02-2022

For 90% dominance:

VoC	EU	alpha	delta	Omicron
Start	01-06-2020	01-02-2021	01-07-2021	31-12-2021
end	04-12-2020	19-05-2021	01-12-2021	01-02-2022

Statistical analysis

SAR was estimated using generalized estimating equations predicting a binary outcome indicating if the contact was infected by the index or not (Contact_infected). The clusters considered were the index cases, and we assumed an exchangeable correlation structure. We used a Gaussian identity link [81], which allows to estimate the relative proportion increase provoked by each covariate relative to a reference proportion of infected contacts, that is the SAR. The estimation equation reads:

Contact_infected ~ immune_index + immune_contact + age_index + age_contact + gender_index + gender_contact + bmi_index + symptoms_index + cough_index + building_type_index + SEC_index + vulnerability_index + contact_type + testing_propensity_contact

Where immune_index is a categorical variable providing the immune status of the index case, immune_contact the one for the contact, age_index and age_contact are the age of the index and contact, gender_index and gender_contact a categorical variable giving the gender of the index and contact, bmi_index the variable describing the body mass index (BMI) of the index, symptoms_index a binary variable indicating the presence of symptoms and cough_index of cough for the index, building_type_index the type of building in which the index lives, SEC_index a categorical variable describing the neighbourhood socio-economic condition of the index, vulnerability_index a binary variable describing the personal vulnerability of the index, contact_type a categorical variable providing the type of relationship between index and contact, and testing_propensity_contact a categorical variable describing the propensity of the contact to test.
