# Supplementary material for: Effect of SARS-CoV-2 prior infection and mRNA vaccination on contagiousness and susceptibility to infection
Source: Nat Commun. 2023 Sep 6;14:5452. doi: 10.1038/s41467-023-41109-9 (PMC10482859; doi:10.1038/s41467-023-41109-9)
Supplement: Supplementary file 3 — Reporting Summary [file 41467_2023_41109_MOESM3_ESM.pdf]

nature portfolio

Corresponding author(s): Denis Mongin

Last updated by author(s): Jul 11, 2023

Reporting Summary

Nature Portfolio wishes to improve the reproducibility of the work that we publish. This form provides structure for consistency and transparency in reporting. For further information on Nature Portfolio policies, see our Editorial Policies and the Editorial Policy Checklist.

Statistics

For all statistical analyses, confirm that the following items are present in the figure legend, table legend, main text, or Methods section.

n/a	Confirmed	
☐	☒	The exact sample size (*n*) for each experimental group/condition, given as a discrete number and unit of measurement
☐	☒	A statement on whether measurements were taken from distinct samples or whether the same sample was measured repeatedly
☐	☒	The statistical test(s) used AND whether they are one- or two-sided<br>*Only common tests should be described solely by name; describe more complex techniques in the Methods section.*
☐	☒	A description of all covariates tested
☐	☒	A description of any assumptions or corrections, such as tests of normality and adjustment for multiple comparisons
☐	☒	A full description of the statistical parameters including central tendency (e.g. means) or other basic estimates (e.g. regression coefficient) AND variation (e.g. standard deviation) or associated estimates of uncertainty (e.g. confidence intervals)
☐	☒	For null hypothesis testing, the test statistic (e.g. F, t, r) with confidence intervals, effect sizes, degrees of freedom and P value noted<br>*Give P values as exact values whenever suitable.*
☒	☐	For Bayesian analysis, information on the choice of priors and Markov chain Monte Carlo settings
☒	☐	For hierarchical and complex designs, identification of the appropriate level for tests and full reporting of outcomes
☐	☒	Estimates of effect sizes (e.g. Cohen's *d*, Pearson's *r*), indicating how they were calculated

Our web collection on statistics for biologists contains articles on many of the points above.

Software and code

Policy information about availability of computer code

Data collection	Data stem from an operational register. The data of the ARGOS register was collected using mainly the REDCap software in conjonction with R scripts.
Data analysis	Code for data management, data analysis and production of tables and figures have been made available at a open gitlab repository : https://gitlab.com/dmongin/scientific_articles/-/tree/main/Effect_of_mRNA_vaccination

For manuscripts utilizing custom algorithms or software that are central to the research but not yet described in published literature, software must be made available to editors and reviewers. We strongly encourage code deposition in a community repository (e.g. GitHub). See the Nature Portfolio guidelines for submitting code & software for further information.

Data

Policy information about availability of data

All manuscripts must include a data availability statement. This statement should provide the following information, where applicable:

- Accession codes, unique identifiers, or web links for publicly available datasets
- A description of any restrictions on data availability
- For clinical datasets or third party data, please ensure that the statement adheres to our policy

Due to privacy issues, these individual-level Data are available upon request at https://edc.hcuge.ch/surveys/?s=TLT9EHE93C. Response is provided within two weeks. Data are provided de-identified and thus exact address is not available.

Research involving human participants, their data, or biological material

Policy information about studies with human participants or human data. See also policy information about sex, gender (identity/presentation), and sexual orientation and race, ethnicity and racism.

Reporting on sex and gender	The study consider gender in the main analysis. Gender was self-reported by the patients. We also considered the same analysis stratified per gender, to account for potential interaction effects.
Reporting on race, ethnicity, or other socially relevant groupings	The study did not record nor use race, ethnicity or other relevant grouping.
Population characteristics	The population is composed of persons with a positive SARS-CoV-2 PCR test and their declared contacts who did not refuse to share their data. The total population considered is of 50973 index cases (mean age of 35.7 years (SD 18.4), 53.9% are women) and 111674 contacts (mean age of 31.4 years (SD 20.1), 51.7% are women).
Recruitment	All persons with a positive SARS-CoV-2 test was collected in this register. From February 2020 to end of April 2020, contact information was collected by interviewing the index case. From May 2020, index cases had the possibility to provide their contacts names and phone through an online form. Contacts were then approached using phone interviews. Additionally, an online form was implemented at the end of September 2020 to support the oral interviews, where the contacts had the ability to complete the required information themselves.<br>All person had the possibility to refuse to share their data.
Ethics oversight	Research received the agreement of the Cantonal Ethic Committee of Geneva (CCER protocol 2020-01273)

Note that full information on the approval of the study protocol must also be provided in the manuscript.

Field-specific reporting

Please select the one below that is the best fit for your research. If you are not sure, read the appropriate sections before making your selection.

☒ Life sciences ☐ Behavioural & social sciences ☐ Ecological, evolutionary & environmental sciences

For a reference copy of the document with all sections, see nature.com/documents/nr-reporting-summary-flat.pdf

Life sciences study design

All studies must disclose on these points even when the disclosure is negative.

Sample size	No sample size calculation was performed, we considered the maximum of data available. Our outcome is Sars-Cov2 infection of the contact, and the multivariable analysis includes 14 factors, corresponding to 29 degree of freedom. In our smallest stratum, we have 3546 events, thus reaching ~122 point per events, far above all recomanded number of point per event for this kind of analysis.
Data exclusions	Individuals who refused to share their data were removed from the analysis.
Replication	To verify the reproducibilty of our findings, we published the entire analysis code (https://gitlab.com/dmongin/scientific_articles/-/tree/main/Effect_of_mRNA_vaccination). The data are available upon request.
Randomization	Due to the retroscpective nature of the study, randomization was not applicable to the study
Blinding	Due to the retroscpective nature of the study, blinding was not applicable to the study

Reporting for specific materials, systems and methods

We require information from authors about some types of materials, experimental systems and methods used in many studies. Here, indicate whether each material, system or method listed is relevant to your study. If you are not sure if a list item applies to your research, read the appropriate section before selecting a response.

Materials & experimental systems

n/a	Involved in the study
☒	☐ Antibodies
☒	☐ Eukaryotic cell lines
☒	☐ Palaeontology and archaeology
☒	☐ Animals and other organisms
☒	☐ Clinical data
☒	☐ Dual use research of concern
☒	☐ Plants

Methods

n/a	Involved in the study
☒	☐ ChIP-seq
☒	☐ Flow cytometry
☒	☐ MRI-based neuroimaging
